# Morphological variation in *Echinorhynchus truttae* Schrank, 1788 and the *Echinorhynchus bothniensis* Zdzitowiecki & Valtonen, 1987 species complex from freshwater fishes of northern Europe

**DOI:** 10.3897/BDJ.1.e975

**Published:** 2013-09-16

**Authors:** Matthew T Wayland

**Affiliations:** † Department of Zoology, University of Cambridge, Cambridge, United Kingdom

**Keywords:** Acanthocephala, *Echinorhynchus truttae*, *Echinorhynchus bothniensis*, *Echinorhynchus leidyi*, cryptic speciation, sibling species, morphology, morphometric, meristic, description, Proboscis Profiler, overdispersion, parasite, salmoniform

## Abstract

*Echinorhynchus truttae* and the *Echinorhynchus bothniensis* species complex are common parasites of salmoniform and other fishes in northern Europe. *Echinorhynchus bothniensis* and its sibling species *Echinorhynchus 'bothniensis'* are thought to be closely related to the Nearctic *Echinorhynchus leidyi* Van Cleave, 1924 based on morphological similarity and common usage of a mysid intermediate host. This study provides the first analysis of morphological and meristic variation in *Echinorhynchus truttae* and expands our knowledge of anatomical variability in the *Echinorhynchus bothniensis* group. Morphological variability in *Echinorhynchus truttae* was found to be far greater than previously reported, with part of the variance attributable to sexual dimorphism. *Echinorhynchus truttae*, the two species of the *Echinorhynchus bothniensis* group and *Echinorhynchus leidyi* displayed considerable interspecific overlap in the ranges of all conventional morphological characters. However, Proboscis profiler, a tool for detecting acanthocephalan morphotypes using multivariate analysis of hook morphometrics, successfully separated *Echinorhynchus truttae* from the other taxa. The *Echinorhynchus bothniensis* species group could not be reliably distinguished from *Echinorhynchus leidyi* (or each other), providing further evidence of the affinity of these taxa. Observations on the distribution of *Echinorhynchus truttae* in its definitive host population are also reported.

## Introduction

*Echinorhynchus bothniensis* Zdzitowiecki & Valtonen, 1987 was originally described from *Osmerus eperlanus* L. from the oligohaline waters of the Bothnian Bay, northern Baltic. In earlier studies (Timola 1980, Valtonen 1980, Valtonen 1983) this acanthocephalan had been determined as *Echinorhynchus gadi* Zoega in Müller, 1776 (see Zdzitowiecki and Valtonen 1987). The first evidence that *Echinorhynchus 'gadi'* in *Osmerus eperlanus* was biologically distinct from *Echinorhynchus gadi* in *Gadus morhua* L. and other marine fish came from amphipod infection experiments; acanthors of *Echinorhynchus gadi* from Baltic *Gadus morhua* were infective to *Gammarus zaddachi* Sexton, but acanthors of *Echinorhynchus 'gadi'* from *Osmerus eperlanus* were not (Valtonen et al. 1983). Moreover, Valtonen et al. (1983) noted that the mature females of *Echinorhynchus 'gadi'* from *Osmerus eperlanus* were smaller than the mature females of *Echinorhynchus gadi* found in *Gadus morhua*. A detailed morphological study by Zdzitowiecki and Valtonen (1987) revealed marked differences in egg dimensions between *Echinorhynchus 'gadi'* from *Osmerus eperlanus* and *Echinorhynchus gadi* from *Gadus morhua*. Furthermore, male *Echinorhynchus 'gadi'* from *Osmerus eperlanus* tended to exhibit one or more pairs of parallel cement glands in contrast to the moniliform pattern displayed by *Echinorhynchus gadi* from *Gadus morhua*, although there was some interspecific overlap in cement gland arrangement. On the basis of these morphological differences Zdzitowiecki and Valtonen (1987) accorded specific rank to *Echinorhynchus 'gadi'* from *Osmerus eperlanus* by naming it *Echinorhynchus bothniensis*. Other true definitive hosts (*i.e.* hosts in which gravid female worms have been found) of *Echinorhynchus bothniensis* from the Bothnian Bay include *Lampetra fluviatilis* (L.), *Salmo trutta* L., *Lota lota* (L.), *Myoxocephalus quadricornis* (L.) and *Platichthys flesus* (L.) (Valtonen and Crompton 1990). The intermediate hosts belongs to the *Mysis relicta* Lovén (Mysidacea) species group (Valtonen and Crompton 1990). It is important to note that **this species group has recently been split, on the basis of molecular and morphological characters (Audzijonytė and Väinölä 2005) into four named taxa: *Mysis relicta* (sensu stricto), *Mysis salemaai* Audzijonyte & Väinölä, 2005, *Mysis segerstralei* Audzijonyte & Väinölä, 2005 and *Mysis diluviana* Audzijonyte & Väinölä, 2005.

Using multilocus enzyme electrophoresis Väinölä et al. (1994) demonstrated that not only is *Echinorhynchus bothniensis* heterospecific to *Echinorhynchus gadi*, but that both taxa represent complexes of sibling species. One species of the *Echinorhynchus bothniensis* group was found in *Osmerus eperlanus* from the Bothnian Bay and in *Osmerus eperlanus* and *Mysis relicta* (sensu stricto) (as *Mysis relicta* sp. I sensu Väinölä 1986) from Lake Keitele, central Finland. Väinölä et al. (1994) referred to this species as *Echinorhynchus bothniensis*, since its range included the type-locality. A second species found in *Coregonus lavaretus* (L.), *Platichthys flesus* (L.), *Salvelinus alpinus* (L.) and *Mysis segerstralei* Audzijonyte & Väinölä, 2005 (as *Mysis relicta* sp. III sensu Väinölä 1986) from Lake Pulmankijärvi, northern Finland was designated *Echinorhynchus 'bothniensis'* (Väinölä et al. 1994). Neither of the lacustrine populations of the *Echinorhynchus bothniensis* group have previously been studied morphologically.

*Echinorhynchus bothniensis* is morphologically very similar to the North American *Echinorhynchus leidyi* Van Cleave, 1924 (= *Echinorhynchus salvelini* Linkins in Ward & Whipple, 1918 *nec* Schrank, 1788), but apparently differs slightly from the latter species in hook formula and cement gland arrangement (Zdzitowiecki and Valtonen 1987). *Mysis relicta* (sensu lato) is reported as the intermediate host of *Echinorhynchus leidyi* (Prychitko and Nero 1983, Wolff 1984). More precisely, these intermediate host records for Nearctic *Echinorhynchus leidyi* will correspond to *Mysis segerstralei* and/or *Mysis diluviana*; *Mysis relicta* sensu stricto appears to be confined to north European fresh and brackish waters (Audzijonytė and Väinölä 2005). Definitive hosts include salmonid and coregonid fishes. Väinölä et al. (1994) postulated that the common usage of *Mysis relicta* group species as intermediate hosts defines *Echinorhynchus leidyi* and the *Echinorhynchus bothniensis* group as a clade of closely related species, because the known life cycles of all other *Echinorhynchus* spp. involve an amphipod intermediate host. Furthermore, these authors advanced an hypothesis of co-speciation of the acanthocephalans with their mysid hosts.

Both *Echinorhynchus bothniensis* and *Echinorhynchus leidyi* have a similar hooks formula and cement gland arrangement to a congener, *Echinorhynchus truttae* Schrank, 1788 found in salmoniform fishes of the Palaearctic. *Echinorhynchus truttae* utilises an amphipod (*Gammarus pulex* (L.)), rather than a mysid, as an intermediate host (Awachie 1966) and so is apparently biologically distinct from the *Echinorhynchus bothniensis* group and *Echinorhynchus leidyi*. Zdzitowiecki and Valtonen (1987) reported that *Echinorhynchus bothniensis* could be distinguished from *Echinorhynchus truttae*, because the latter has a longer proboscis and much shorter eggs. However, the diagnostic value of these characters was equivocal, since anatomical variability in *Echinorhynchus truttae* had never been assessed. The means to discriminate between the *Echinorhynchus bothniensis* group and *Echinorhynchus truttae* is of real significance to workers conducting faunistic surveys or other studies on these acanthocephalans. The two taxa share some of the same species of definitive host (*e.g.Salmo trutta*) (see Petrochenko 1956, Valtonen and Crompton 1990) and may well occur in sympatry, since their intermediate hosts have overlapping geographical ranges in northern Europe (see Pinkster 1978, Väinölä et al. 1994).

*Echinorhynchus truttae* is typically a parasite of salmoniform fishes and has been reported from a variety of species including *Salmo trutta* (*e.g.*Awachie 1966), *Salvelinus alpinus* (*e.g.*Dorucu et al. 1995), *Salvelinus leucomaenis* (Pallas) (Nagasawa et al. 1997), *Coregonus lavaretus* (*e.g.*Petrochenko 1956), *Thymallus thymallus* (L.) (e.g. Petrochenko 1956), *Thymallus arcticus baicalensis* Dybowski (Baldanova and Pronin 1998, Baldanova 2000) and *Oncorhynchus mykiss* (Walbaum) (Dorucu et al. 1995, Holland and Kennedy 1997). *Echinorhynchus truttae* is found throughout Europe (including Ireland and the British Isles) and its range extends across Siberia all the way to the Bering Straits (Petrochenko 1956). Golvan (1994) suggested that *Echinorhynchus truttae* (sensu Zhukov 1960) from the Kurile Islands, northwest Pacific, may be another species.

The principal aims of the present study were: (i) to ascertain whether the two sibling species of the *Echinorhynchus bothniensis* group can be distinguished from each other, and from *Echinorhynchus leidyi*, using morphological characters; (ii) to review the taxonomy of *Echinorhynchus bothniensis* and *Echinorhynchus 'bothniensis'*; (iii) to quantify morphological variability in *Echinorhynchus truttae*; and (iv) to identify the best characters for discriminating this taxon from the *Echinorhynchus bothniensis* sibling species and *Echinorhynchus leidyi*. Additionally, some observations on the ecology of *Echinorhynchus truttae* are reported.

## Material and methods

### Material

Table 1 provides a detailed list of all material studied, including accession numbers. A total of 19 specimens (7 males; 12 females) of *Echinorhynchus truttae* were collected from wild brown trout (*Salmo trutta* L.) from two streams (Loch Walton Burn and Loch Coulter Burn) in the River Carron catchment, central Scotland. The fish were caught by electro-fishing and were transported live to the laboratory where they were killed by a blow to the head and examined for acanthocephalan infection within 24 hours. Acanthocephalans found were washed and relaxed in refrigerated distilled water before being fixed in 75% alcohol. These acanthocephalans were identified as *Echinorhynchus truttae* using the keys in Petrochenko (1956). They were judged to be *Echinorhynchus truttae*, rather than members of the morphogically similar *Echinorhynchus bothniensis* group or *Echinorhynchus leidyi*, because the lotic environment they were collected from is unlikely to support populations of the lentic *Mysis relicta*, the intermediate host of the *Echinorhynchus bothniensis* group. Furthermore, the trout sampled were in their first year of life and so were unlikely to have spent any time outside their natal stream where they might potentially have been infected with *Echinorhynchus bothniensis*.

A series of *Echinorhynchus truttae* (74 specimens; 45 females; 29 males) collected by Dr A Pike, University of Aberdeen, from *Salmo trutta* from Drummore, on the south-west coast of Scotland, held in the spirit collection of the Natural History Museum was also studied. Most of these acanthocephalans had well everted probosces and displayed no tegumental folding, suggesting that they had been relaxed in water before being fixed.

All of the specimens of the *Echinorhynchus bothniensis* group studied were collected between 1985 and 1997 by Professor E. T. Valtonen of the University of Jyväskylä and deposited in the spirit collection of The Natural History Museum. Some of this material had been fully relaxed in water prior to fixation. Most of the *Echinorhynchus bothniensis* material came from one host species, *Osmerus eperlanus*, from the freshwater Lake Keitele, central Finland. This population of *Echinorhynchus bothniensis* is thought to have been isolated from conspecifics in the Bothnian Bay for at least 6,000 years (Väinölä et al. 1994). Five paratypes of *Echinorhynchus bothniensis* (BM(NH) 1987.1070-1074) from *Osmerus eperlanus* from the Bothnian Bay were also examined, but only one female worm was in a suitable condition for measuring hook morphometrics.

*Echinorhynchus 'bothniensis'* is known only from Lake Pulmankijärvi in northern Lapland, on the Finnish-Norwegian border. This freshwater lake lies 17 metres above sea level and drains into the Barents Sea. Samples of *Echinorhynchus 'bothniensis'* were obtained from the following hosts: *Salvelinus alpinus* (L.), *Coregonus lavaretus* (L.) and *Platichthys flesus* (L.).

In addition to the northern European material described above, voucher specimens of the Nearctic *Echinorhynchus leidyi* from the Canadian Museum of Nature were also examined. These acanthocephalans were collected by Shostak et al. (1986) during their extensive survey of morphological variability in *Echinorhynchus gadi*, *Echinorhynchus leidyi* and *Echinorhynchus salmonis* Müller, 1784 from northern Canada.

### Light Microscopy

The specimens of *Echinorhynchus leidyi* from the Canadian Museum of Nature had been fixed in formalin-acetic acid-alcohol (FAA), stained with Semichon's carmine and permanently mounted in Permount (Fisher Scientific). All other acanthocephalans were prepared for light microscopy by dehydration through an alcohol series followed by clearing in lactophenol. Measurements were made with aid of a digitizing tablet (KS 100, Version 3, Carl Zeiss Vision). Hook morphometric data were recorded from one longitudinal row in which all of the hooks were visible in profile using the method described by Wayland (2010). Morphometric and meristic data were collected during a PhD studentship (Wayland 2002).

### Morphological Data Analysis

Statistical analysis and visualization of morphometric and meristic data were performed using the R language and environment (R Core Team 2012). Boxplots augmented with strip charts were created using the R package beeswarm (Eklund 2012). Proboscis profiler (Wayland 2010) was used to analyse both intra and interspecific variation in hook measurements. Proboscis profiler, based on the meristogram of Huffman and Bullock (1975), was developed to detect morphological heterogeneity in collections of superficially similar acanthocephalan worms based on the multivariate statistical analysis of proboscis hook dimensions. For a detailed description of this tool with examples, please refer to Wayland (2010). In brief, the Proboscis profiler algorithm is composed of the following sequential steps:

1. Proboscis profiler requires blade length and base width measurements from each of the hooks in at least one longitudinal row of hooks per specimen. In each longitudinal row hooks are numbered sequentially, starting with the most distal hook.
2. Hook position is standardized. Counted position number of each hook in a given row is multiplied by 100 and divided by n + 1, where n = the total number of hooks in the row and the constant 1 is a corrective factor for centring the data-points in graphs.
3. A moving average (arithmetic mean) routine is applied to the data from each row of hooks and considers a user-defined segment of the percent-position axis for each measurement (length and base). The segment advances through the data from anterior to posterior in 1% increments.
4. Unsupervised pattern recognition using principal component analysis.
5. Hierarchical clustering of the first two principal components from step 4.

### Ecological Data Analysis

For each of the two host populations studied (Loch Walton Burn and Loch Coulter Burn), Quantitative Parasitology (Rózsa et al. 2000, Reiczigel 2003) was used to calculate an exact confidence interval for the prevalence of infection (using the Sterne method), a bootstrap confidence interval for mean abundance and the aggregation index (variance/mean). The R package fitdistrplus (Delignette-Muller et al. 2013) was used to determine whether a Poisson or a negative binomial distribution provided the best description of the occurrence of *Echinorhynchus truttae* in its definitive host populations.

## Data resources

All data collected for this study are available as supplementary files.

### Morphological data

Standard morphometric and meristic data for female and male acanthocephalans can be found in Suppl. materials 1, 2 respectively. Egg and acanthor dimensions are listed in Suppl. material 3. Hook measurement data for female and male acanthocephalans (Suppl. materials 4, 5 respectively) are in a file format suitable as input to the Proboscis Profiler software (Wayland 2010).

### Ecological data

Suppl. materials 6, 7 contain data on the occurrence of *Echinorhynchus truttae* in samples of its definitive host *Salmo trutta* from Loch Coulter and Loch Walton respectively. For each fish examined, fork length and intensity of infection were recorded.

## Results

### Variation in conventional morphological characters

Initially an assessment was made of intraspecific and interspecific variation in conventional morphological characters, *i.e.* those characters used by most acanthocephalan taxonomists in the differential diagnosis of *Echinorhynchus* species. Summaries of these variables for the female and male acanthocephalans examined in this study are provided in Tables 2, 3 respectively. Data for the three *Echinorhynchus truttae* populations (Loch Walton Burn, Loch Coulter Burn and Drummore) have been pooled, because, in the absence of any inter-site morphological variability, these acanthocephalans were assumed to be conspecific. Additionally, for comparative purposes, Tables 2, 3 contain data for *Echinorhynchus bothniensis* from *Osmerus eperlanus* in the Bothnian Bay (original description by Zdzitowiecki and Valtonen 1987) and an extensive collection of *Echinorhynchus leidyi* from various fishes across northern Canadian waters (Shostak et al. 1986). It is important to note that these additional data were recorded from acanthocephalans prepared for light microscopy using methods different from those employed in the current study, although in all studies acanthocephalans were relaxed in fresh water prior to fixation to evert proboscides. Zdzitowiecki and Valtonen (1987) fixed their samples of *Echinorhynchus bothniensis* in alcohol and examined them as wet mounts, similarly to the current study, however they used creosote rather than lactophenol as a clearing agent. By contrast, Shostak et al. (1986) fixed their samples in formalin-acetic acid-alcohol (FAA), stained them with acetocarmine and mounted them in synthetic resin.

The extent of intraspecific morphological variability for the taxa studied can be seen in Tables 2, 3. The mean and range of values for each morphometric are very similar for both *Echinorhynchus bothniensis* population, i.e. the Bothnian Bay and Lake Keitele. An analysis of the cause of intraspecific variation in morphological characters was attempted for *Echinorhynchus truttae* only, as sample numbers for the other taxa were considered to be too small for a meaningful statistical analysis. All anatomical characters common to both sexes are larger in females than males (compare data in tables Tables 2, 3 and also see boxplots in Suppl. material 8). Sexual dimporphism is also clearly apparent in a principal components analysis of conventional morphological characters (Fig. 1a). There is considerable separation of females from males in the first principal component, which accounts for 36% of the variation in the dataset. The variables contributing most to the separation of the two sexes (i.e. those with the highest loadings for principal component one) are: lemniscus length, proboscis receptacle length and width, body length and proboscis length and width (Fig. 1b). Body size is positively correlated (Bonferroni corrected p-value < 0.05) with the size of several anatomical characters of female *Echinorhynchus truttae* (Table 4), namely, body width (r^2^=0.257), proboscis length (r^2^=0.317), proboscis receptacle length (r^2^=0.284), lemniscus length (r^2^=0.364), lemniscus width (r^2^=0.237), vagina width (r^2^=0.246) and vaginal sphincter width (r^2^=0.251). In male *Echinorhynchus truttae* (Table 5), a significant positive correlation with body length is only demonstrated for the length of the reproductive system (r^2^= 0.876), lemniscus length (r^2^=0.487) and the length of the testes (r^2^=0.346 for anterior testis; r^2^=0.469 for posterior testis). Evidence of morphological variation in *Echinorhynchus truttae* between the three sample sites was not found, even after taking sexual dimorphism into account.

Although there are interspecific differences in the means of some of the morphometric variables (*e.g.* maximum length of hook blade) listed in Tables 2, 3, interspecific overlap in their ranges prevents any single morphometric variable from being used to reliably discriminate any of the species in this study. For a graphical representation of interspecific variation in each conventional morphological character, see boxplots in Suppl. materials 9, 10.

Marked intraspecific, but subtle interspecific anatomic variation was observed in the male reproductive system. Four of 32 male *Echinorhynchus truttae* had only one testis, which measured 793–1530 × 393–730µm. No monorchid males were found in *Echinorhynchus bothniensis* or *Echinorhynchus 'bothniensis'*. All of the *Echinorhynchus* spp. studied typically displayed six cement glands, but the number of glands was variable in *Echinorhynchus 'bothniensis'* and *Echinorhynchus truttae*. Of eleven specimens of *Echinorhynchus 'bothniensis'*, nine (82%) exhibited six cement glands, but two (18%) had only five. Cement gland number was recorded from 35 male *Echinorhynchus truttae*; the numbers displaying 4, 5, 6 and 8 cement glands were 1 (3%), 3 (9%), 30 (86%) and 1 (3%), respectively. Cement gland arrangements of specimens with six glands are summarized in Table 6. It is interesting to note that none of the specimens of *Echinorhynchus truttae* were found to exhibit the moniliform pattern (chain-like, six singles) and that the majority (96%) had either one or two paired cement glands. This is in contrast to the other taxa, where a large proportion of the males (21–57%) display the moniliform pattern. In *Echinorhynchus 'bothniensis'* pairs of cement glands consisted of the third and fourth, or fourth and fifth glands from the anterior. In *Echinorhynchus bothniensis* pairs were made up of any two adjacent cement glands (*i.e.* first and second, second and third, third and fourth, fourth and fifth or fifth and sixth).

### Proboscis profiles

Before attempting to use the Proboscis Profiler to discriminate taxa, potential confounding variables should be considered. Preparation is one such problem (Palaearctic samples fixed in alchol, then cleared and temporarily mounted in lactophenol *vs* Nearctic samples fixed in FAA, stained with acetocarmine and permanently mounted in synthentic resin), but cannot be controlled in this analysis. Therefore, it is important to exercise caution when making comparisons between *Echinorhynchus leidyi* and the other taxa. Radial asymmetry of proboscis hooks is another potential problem (Wayland 2010). Unfortunately, the importance of radial asymmetry was not known at the time of data collection and so no record was made of which surface of the proboscis (dorsal, ventral or lateral) the measured hooks were situated. One confounding factor which can be measured and, if necessary, controlled (by profiling females and males separately) is sexual dimorphism. This phenomenon was investigated in *Echinorhynchus truttae*, because hook data from a complete longitudinal row are available (Suppl. materials 4, 5) for a relatively large number of both female (n=46) and male (n=26) acanthocephalans.

Fig. 2 shows hook blade length and base width variables of the 72 *Echinorhynchus truttae* specimens plotted against a standardized position (for definition, see morphological data analysis section of material and methods). Sexual dimorphism is not readily apparent in these two plots. Proboscis profiles were generated with a moving average segment of 11; the minimum sized moving average segment that can be applied to this dataset. Principal component analysis of these proboscis profiles revealed subtle sexual dimorphism, with some separation of the females from males in principal component one (PC1), which describes 49% of the variation in the dataset (Fig. 3a). A Welch two sample t-test found a significant difference (p=0.005) between females and males in the scores for PC1. Base width variables show higher loadings than blade length variables for PC1 (Fig. 3b), suggesting that female *Echinorhynchus truttae* tend to have 'stouter' hooks than males. In view of this strong evidence of sexual dimorphism in proboscis profiles, the two sexes are considered separately in the inter-specific comparisons that follow.

**Figure 6.**
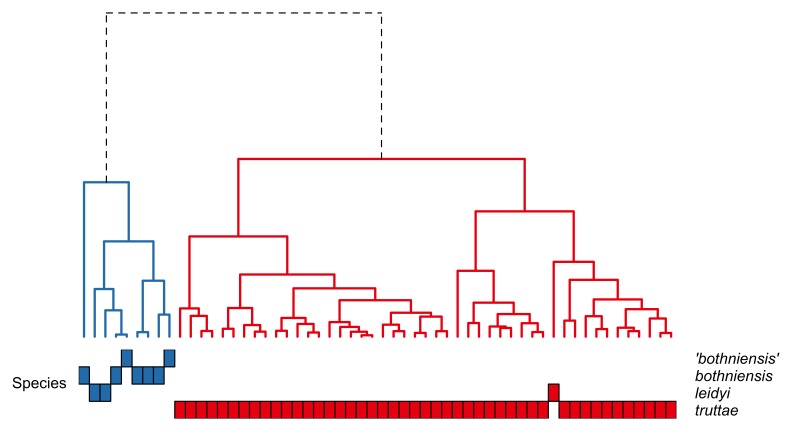
Dendrogram showing the similarity between the proboscis profiles of female *Echinorhynchus bothniensis*, *Echinorhynchus 'bothniensis'*, *Echinorhynchus leidyi* and *Echinorhynchus truttae*. A principal component analysis was applied to the proboscis profile data and the dendrogram was created from hierarchical clustering of the scores for principal components one and two. Analysis based on data in Suppl. material 4.

Proboscis profiles for 56 female acanthocephalans (5 of *Echinorhynchus bothniensis*, 2 of *Echinorhynchus 'bothniensis'*, 3 of *Echinorhynchus leidyi* and 46 of *Echinorhynchus truttae*) were generated using a moving average segment of 10; the minimum sized moving average segment applicable. This dataset of female hook morphometrics (Suppl. material 4) includes data from one of the paratypes of *Echinorhynchus bothniensis* from the Bothnian Bay. Fig. 4 shows positional variation in raw hook morphometrics of female worms; whilst some interspecific variation is apparent, the taxa are indistinguishable. A principal component analysis of the proboscis profiles was performed and a scatterplot of the scores for the first two principal components (Fig. 5a) shows a clear separation of *Echinorhynchus truttae* from the other taxa. The loadings plot for the first two principal components (Fig. 5b) shows that blade length and base width measurements from hooks in the 80.5–95.5% region of the proboscis are driving the separation of *Echinorhynchus truttae* from the other taxa along PC1 (this first principal component accounts for 64% of the variance in the dataset). *Echinorhynchus bothniensis*, *Echinorhynchus 'bothniensis'* and *Echinorhynchus leidyi* are not separated from each other in the scores plot for PC1 and PC2. Hierarchical clustering was used to objectively partition the proboscis profiles into morphotypes; a Euclidean distance matrix was calculated from the scores for PC1 and PC2 and a dendrogram was computed using the complete agglomeration method as implemented in the R function hclust (Fig. 6). The dendrogram shows the presence of two distinct groups: one containing all profiles of *Echinorhynchus truttae* and the other comprising the profiles of the other taxa. The proboscis profile of one specimen of *Echinorhynchus leidyi* clustered with the *Echinorhynchus truttae* profiles. The *Echinorhynchus truttae* cluster comprises two subclusters which are not related to geographical location.

**Figure 9.**
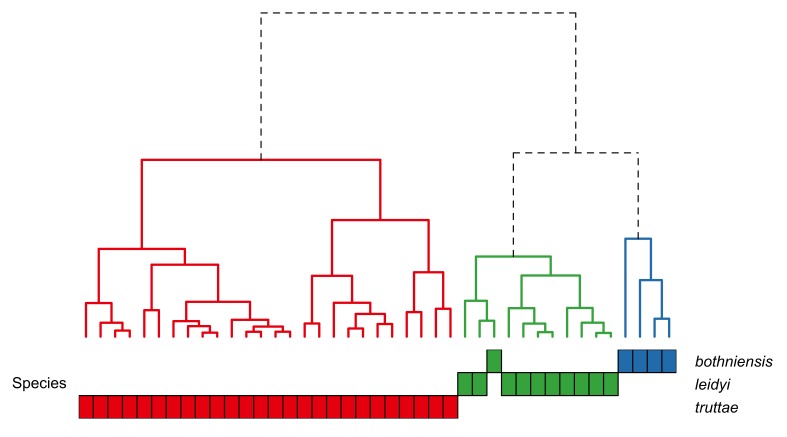
Dendrogram showing the similarity between the proboscis profiles of male *Echinorhynchus bothniensis*, *Echinorhynchus leidyi* and *Echinorhynchus truttae*. A principal component analysis was applied to the proboscis profile data and the dendrogram was created from hierarchical clustering of the scores for principal components one and two. Analysis based on data in Suppl. material 5.

None of the male specimens of *Echinorhynchus 'bothniensis'* had fully everted proboscides and so hook morphometric data could not be collected from them. Therefore, the analysis of interspecific variation in proboscis profiles for male worms was limited to three species: *Echinorhynchus bothniensis* (n=5), *Echinorhynchus leidyi* (n=10) and *Echinorhynchus truttae* (n=26) (data available as Suppl. material 5). Plots of hook morphometrics against standardized position (Fig. 7) show some separation of *Echinorhynchus truttae* from the other taxa; this is most apparent in blade length measurements towards the base of the proboscis (Fig. 7b). Proboscis profiles were generated with a moving average segment of 11, the minimum applicable to the dataset, and then further investigated using principal components analysis. A scores plot for PC1 and PC2 (Fig. 8a) showed a clear separation of *Echinorhynchus truttae* from the other two taxa, and a partial separation of *Echinorhynchus bothniensis* from *Echinorhynchus leidyi*. As was found for the female proboscis profiles, blade length and base width measurements from hooks at the base of the proboscis (80–95% region) are driving the separation of *Echinorhynchus truttae* from the other taxa (Fig. 8b). Hierarchical clustering partioned the male proboscis profiles into three groups corresponding to the three taxa (Fig. 9). However, the proboscis profiles for one of the 10 speciemens of *Echinorhynchus leidyi* was placed in the *Echinorhynchus bothniensis* cluster. As in the dendrogram for female acanthocephalans, the *Echinorhynchus truttae* branch bifurcates into two subclusters which are not related to sampling locality.

### Ecological observations

The frequency distribution of *Echinorhynchus truttae* in its definitive host *Salmo trutta* was recorded for two localities: Loch Walton Burn and Loch Coulter Burn (summary statistics in Table 7; raw data available in Suppl. materials 6, 7). Prevalence of infection was low in both host populations, as were the mean and maximum intensity of infection. Nevertheless, the acanthocephalans were successfully mating, as evident from the presence of gravid females in fish from both localities. The aggregation index was greater than unity in both localities, indicating that the acanthocephalans were overdispersed in their host populations. To further investigate the frequency distribution of the parasite in its host populations, two theoretical distributions were fitted to each dataset (Fig. 10); the Poisson distribution is a good model for a random distribution, while the negative binomial describes overdispersion. A chi-squared test showed that a fitted negative binomial distribution was not significantly different from the observed distribution at both localities (Loch Walton, chi-squared statistic 2.03, p-value 0.155; Loch Coulter, chi-squared statistic 1.81, p-value 0.178). Conversely, the Poisson distribution was a poor fit to the observed data (Loch Walton, chi-squared statistic 13.2, p-value 0.00135; Loch Coulter, chi-squared statistic 6.13, p-value 0.0467).

*Gammarus pulex*, the intermediate host of *Echinorhynchus truttae*, was abundant in both streams. One hundred specimens of this amphipod from Loch Walton Burn were examined by dissection, and while no larval *Echinorhynchus truttae* were found, four cystacanths of *Polymorphus minutus* (Goeze, 1782) (Polymorphida: Polymorphidae) were encountered.

## Discussion

### Intraspecific morphological variation

This study provides the first detailed account of morphometric and meristic variation in adult *Echinorhynchus truttae*, albeit for populations within a small part of its known geographical range. In the absence of evidence to the contrary, the *Echinorhynchus truttae* samples are assumed to comprise a single biological species. However, given the ubiquity of cryptic speciation in the Acanthocephala (Buron et al. 1986, Väinölä et al. 1994, Steinauer et al. 2007, Martínez-Aquino et al. 2009), this assumption might be unwarranted. The *Echinorhynchus truttae* material examined in the present study conforms well to other published descriptions (Lühe 1911, Meyer 1933, Hoffman 1954) but displays considerably greater morphological variability. The only notable difference between the descriptions provided by different authors concerns the size of the eggs. The wide range of egg dimensions recorded in the present study (120–173 × 22–34 µm) ecompasses the measurements reported by Hoffman (1954) (138 × 24 µm), but not the range of dimensions reported by Lühe (1911) (100–110 × 23–24 µm) and Meyer (1933) (100–110 × 24 µm). Discrepancies in egg dimensions between different studies are most likely the result of different fixatives and clearing agents being used to prepare the material for light microscopy, but may also be due to differences in the state of maturity of the acanthors. Shrinkage of eggs following fixation, staining and mounting has been reported by many authors (*e.g.*Lynch 1936, Cleave and Timmons 1952, Cable and Hopp 1954, Bullock 1962).

*Echinorhynchus truttae* exhibited sexual dimorphism in all morphometric variables common to both genders. Within each gender, a proportion of the variance in some morphometric variables was explained by body length. Seven morphometric variables (body width, proboscis length, proboscis receptacle length, lemniscus length and width, vagina width and vaginal sphincter width) were found to be positively correlated with body length in female worms, whilst just four (length of reproductive system, lemniscus length, length of both anterior and posterior testis) showed this relationship in males. However, the length range and sample size of male worms was small relative to that of females and this would have made it more difficult to find evidence of any correlation. A positive correlation with body length can be demonstrated for the size of most anatomical structures in palaecanthocephalans (*e.g.*Amin and Redlin 1980, Brown 1987). Awachie (1966) found that both female and male *Echinorhynchus truttae* increase in length with time spent in the intestine of their definitive host, *Salmo trutta*, and that proboscis length increases with body size. Furthermore, body length and time spent in the definitive host intestine were also positively correlated with sexual maturation in female worms.

Proboscis profiler provided tentative evidence for the presence of two distinct morphotypes within *Echinorhynchus truttae* (Figs 6, 9). This variation was not related to geography, as both subgroups contained samples from both the River Carron catchment, central Scotland and Drummore, southwest Scotland. A molecular genetic analysis would be required to test the hypothesis that these two apparent morphotypes represent sibling species.

Small sample sizes prohibited a statistical analysis of intraspecific morphological variation in the other taxa studied. However, comparison of the mean values and ranges of most morphometric variables (Tables 2, 3) suggest that these taxa also display sexual dimorphism. The Bothnian Bay and Lake Keitele populations of *Echinorhynchus bothniensis* are thought to have been reproductively isolated for at least 6000 years (Väinölä et al. 1994); however, this study did not find any obvious morphological divergence between them.

### Discrimination of species using morphological characters

The genetic differentiation of *Echinorhynchus bothniensis* and *Echinorhynchus 'bothniensis'* into distinct biological species, as evidenced from allozyme electrophoresis (Väinölä et al. 1994), was not accompanied by obvious divergence in conventional morphological characters. Furthermore, proboscis profiler failed to discriminate these species on the basis of female hook morphometrics. Proboscis profiler could not be used to compare the males of these species, as hook data were not available for male *Echinorhynchus 'bothniensis'*. Proboscis profiler has been used to successfully discriminate two species of the *Echinorhynchus gadi* species group identified by allozyme electrophoresis (Wayland 2010). However, *Echinorhynchus bothniensis* and *Echinorhynchus 'bothniensis'* probably diverged more recently than the sibling species of the *Echinorhynchus gadi* group (Väinölä et al. 1994) and therefore have had less time to undergo adaptive morphological change. Moreover, if *Echinorhynchus bothniensis* and *Echinorhynchus 'bothniensis'* occur in allopatry, but utlise similar intermediate and definitive hosts, there may be little or no selection pressure to drive morphological divergence. In contrast, the sibling species of *Echinorhynchus gadi* separable by Proboscis profiler occur in sympatry and often in the same host individual. In this case, adaptation to different regions of the definitive host intestine to avoid competition and/or hybridization may have resulted in anatomical changes to the hooks of the proboscis (Wayland et al. 2005).

The anatomically similar *Echinorhynchus leidyi* from the Nearctic has not been investigated using molecular markers and so its systematic homogeneity and relationship to *Echinorhynchus bothniensis* and *Echinorhynchus 'bothniensis'* may only be speculated. *Echinorhynchus leidyi* could not be discriminated from *Echinorhynchus bothniensis* or *Echinorhynchus 'bothniensis'* using any conventional morphological character or the proboscis profiles of female worms. When applied to male worms, proboscis profiler was quite successful in separating four specimens of *Echinorhynchus bothniensis* from ten specimens of *Echinorhynchus leidyi*, however a fifth specimen of *Echinorhynchus bothniensis* was assigned to the *Echinorhynchus leidyi* cluster (Fig. 9). Nevertheless, this observation should be interpreted with caution as it is based on a small sample of acanthocephalans and may be an artifact of the different protocols used to prepare samples of the two taxa for light microscopy.

The inability of multivariate statistical analysis to reliably distinguish the Nearctic *Echinorhynchus leidyi* from the Palaearctic *Echinorhynchus bothniensis* and *Echinorhynchus 'bothniensis'*, on the basis of morphological characters, is further evidence of the phylogenetic affinity of these taxa. If these acanthocephalans have co-speciated with their mysid intermediate hosts, as hypothesised by Väinölä et al. (1994), they will be members of a clade comprising at least four sibling species (Audzijonytė and Väinölä 2005), some of which may occur in sympatry and at least one may have a circumarctic distribution. An extensive sampling effort combined with tandem molecular and morphological analysis was needed to differentiate and characterize the species of the *Mysis relicta* (sensu lato) group; a similar strategy will be required to investigate the diversity in their echinorhynchid parasites.

*Echinorhynchus truttae* could not be discriminated from *Echinorhynchus leidyi* and the *Echinorhynchus bothniensis* species complex on the basis of any single conventional morphological character. However, Proboscis profiler successfully separated *Echinorhynchus truttae* from *Echinorhynchus leidyi*, *Echinorhynchus bothniensis* and *Echinorhynchus 'bothniensis'*. The hook morphometric data available here as supplementary files (Suppl. materials 4, 5) serve as a useful reference for *Echinorhynchus truttae*, *Echinorhynchus leidyi* and the *Echinorhynchus bothniensis* species group, to which new samples of *Echinorhynchus* spp. from fresh and brackish waters can be compared using Proboscis profiler.

### Distribution of acanthocephalans in their definitive host populations

The frequency distribution of macroparasites within their host populations almost invariably shows overdispersion or aggregation; most hosts harbour few or no parasites, and a few hosts harbour large numbers of parasites (Crofton 1971, Pennycuick 1971, Anderson and May 1978, Anderson and Gordon 1982, Dietz 1982, Dobson 1985, Grenfell et al. 1986, Pacala and Dobson 1988, Guyatt and Bundy 1991, Shaw et al. 1998). Overdispersion is described empirically by the negative binomial distribution (Crofton 1971). In the case of natural infections of Acanthocephala, this distribution has previously been shown to provide an accurate description of the following species in their definitive host populations: *Acanthocephalus clavula* (Dujardin, 1845) in *Gasterosteus aculeatus* L. (see Pennycuick 1971) and *Anguilla anguilla* (L.) (see Shaw et al. 1998); *Acanthocephalus lucii* (Müller, 1776) in *Perca fluviatilis* (L.) (see Shaw et al. 1998); and *Echinorhynchus canyonensis* Huffman & Kliever, 1977 in *Maynea californica* Gilbert (see Huffman and Kliever 1977). In this study the negative binomial provided a good model of the distribution of *Echinorhynchus truttae* in two populations of its definitive host *Salmo trutta*. However, Hine and Kennedy (1974) found that the negative binomial was a poor fit to the frequency distribution of *Pomphorhynchus laevis* (Müller, 1776) in *Leuciscus leuciscus* (L.), even though the parasite was not randomly distributed in its host population.

The negative binomial distribution has also been used to quantify aggregation of larval acanthocephalans in populations of their intermediate hosts. Hine and Kennedy (1974) found that it was a good fit to the observed frequency distribution of *Pomphorhynchus laevis* in a population of *Gammarus pulex* (L.). If there is parasite-induced host mortality, as in the case of natural infections of *Gammarus pulex* by *Polymorphus minutus* (Goeze, 1782), then a truncated negative binomial model is more appropriate (Crofton 1971).

Overdispersion of parasites in their host populations may have various causes, including seasonality in the occurrence of infective stages, spatial aggregation of infective stages, and differences between hosts in behaviour, physiology and immune response to the parasites (e.g. Crofton 1971, Pacala and Dobson 1988, Shaw et al. 1998). *Echinorhynchus truttae* is known to display a seasonal pattern of abundance in its intermediate host, *Gammarus pulex* (see Awachie 1966). However, seasonality should only be a cause of overdispersion in data-sets comprising samples taken throughout the year; in this study the two *Echinorhynchus truttae* data-sets each represented single samples.

Aggregation of cystacanths of *Echinorhynchus truttae* in its amphipod intermediate host *Gammarus pulex*, is a potential cause of the acanthocephalan's overdispersion in its definitive host *Salmo trutta*. Since cystacanths of *Polymorphus minutus* and *Pomphorhynchus laevis* have been found to be aggregated in populations of *Gammarus pulex*, then it is plausible that the same phenomenon occurs in *Echinorhynchus truttae*. If the larvae of *Echinorhynchus truttae* were aggregated in their intermediate host population, then, although their fish hosts may have encountered intermediate hosts at random, the worm burden of the intermediate hosts encountered would not be random. This would lead to a heterogenous distribution of acanthocephalans in the fish population.

It is important to note that overdispersion of acanthocephalans in their definitive hosts can occur in the absence of spatial aggregation of cystacanths. Crompton et al. (1984) found that *Moniliformis moniliformis* (Bremser, 1811) Travassos, 1915 (as *Moniliformis dubius* Meyer, 1932) had an aggregated distribution in groups of rats (*Rattus norvegicus* (Berkenhout)) in which every rat had been fed the same number of cystacanths. Valtonen and Crompton (1990) found that the prevalence and overdispersion of *Echinorhynchus bothniensis* infections of *Osmerus eperlanus* increased with host size. This observation suggests that overdispersion in this particular host-parasite system is linked to some aspect of the interaction between parasite and definitive host.

Experimental work is necessary to determine the causes of overdispersion of acanthocephalans in their host populations. *Moniliformis moniliformis* in rats serves as a convenient laboratory model for studies on acanthocephalan dispersion in mammalian host populations (Crompton et al. 1984, Stoddart et al. 1991). *Echinorhynchus truttae* in *Salmo trutta* might represent a useful model for studies of acanthocephalan dispersion in fish populations, since this species has a life cycle which can be completed in the laboratory (Awachie 1966).

## Figures and Tables

**Figure 1a.**
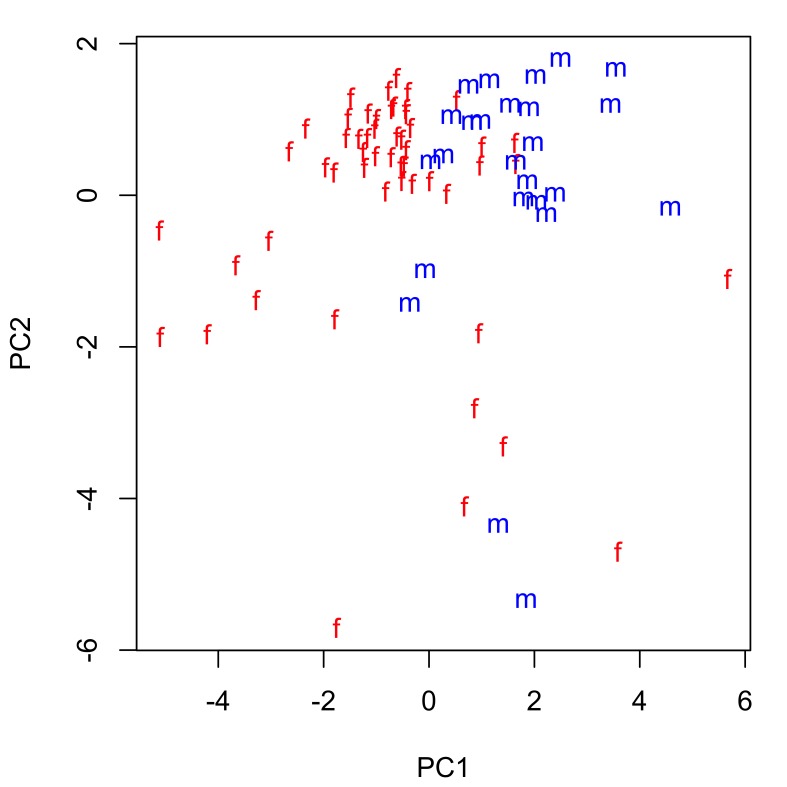
Scatterplot of the scores for the first two principal components (PC1 and PC2). Key: f, female; m, male.

**Figure 1b.**
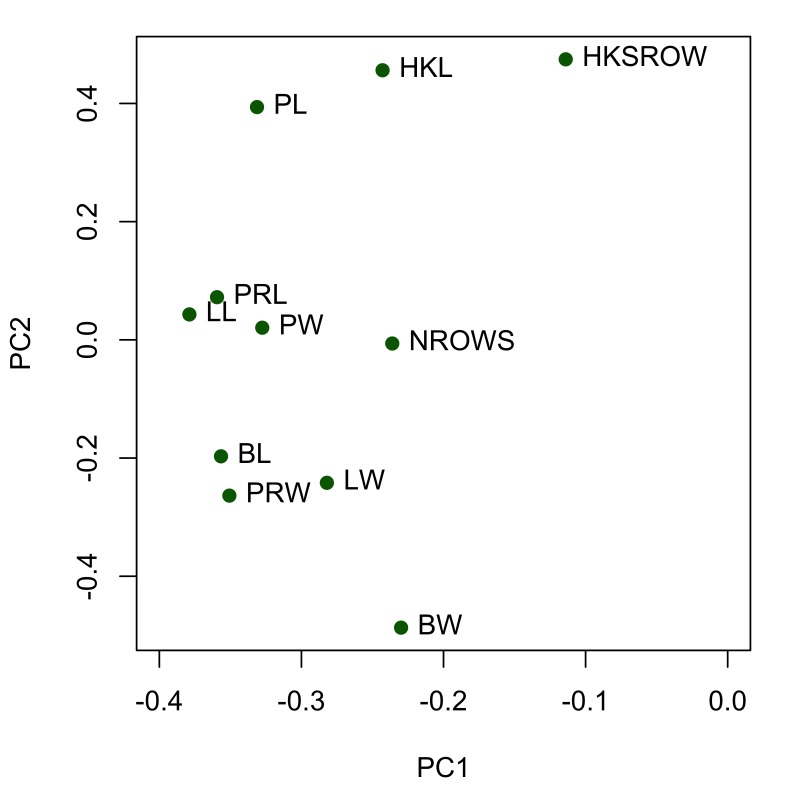
Scatterplot of the loadings for PC1 and PC2. Key: BL, body length; BW, body width; PL, proboscis length; PW, proboscis width; PRL, proboscis receptacle length; PRW, proboscis receptacle width; LL, lemniscus length; LW, lemniscus width; HKL, maximum hook blade length; NROWS, number of longitudinal rows of hooks; HKSROW, maximum number of hooks per longitudinal row.

**Figure 2a.**
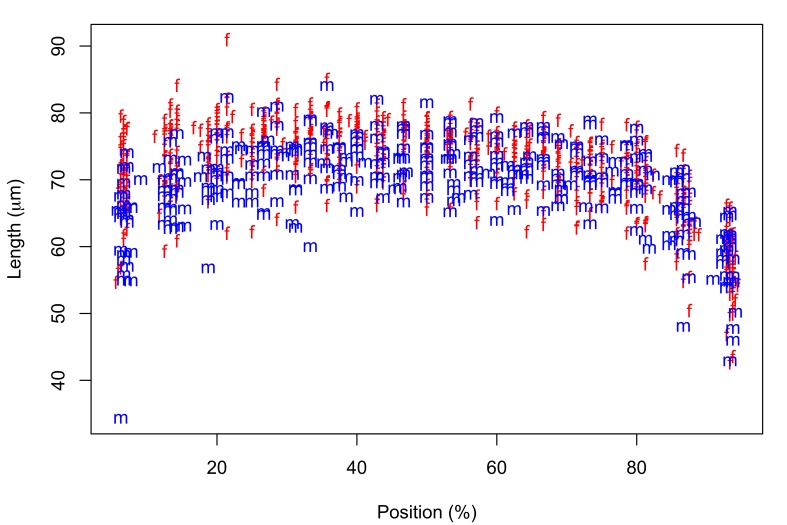
Hook blade length plotted against standardized position.

**Figure 2b.**
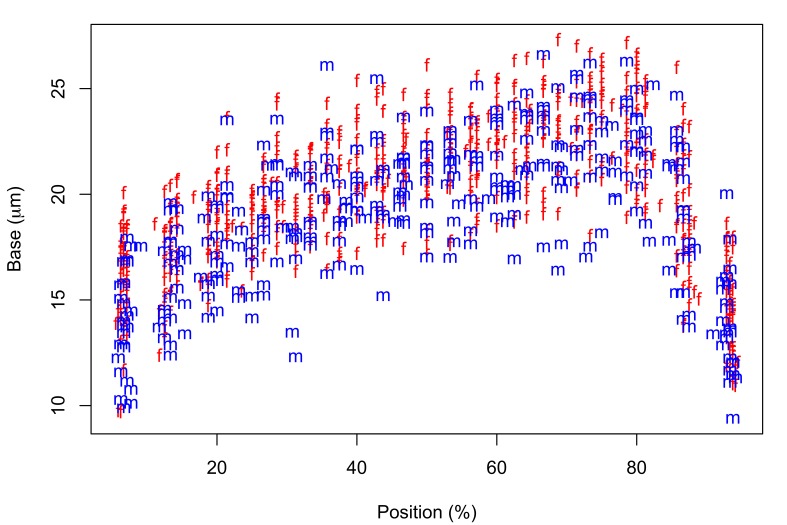
Hook base width plotted against standardized position.

**Figure 3a.**
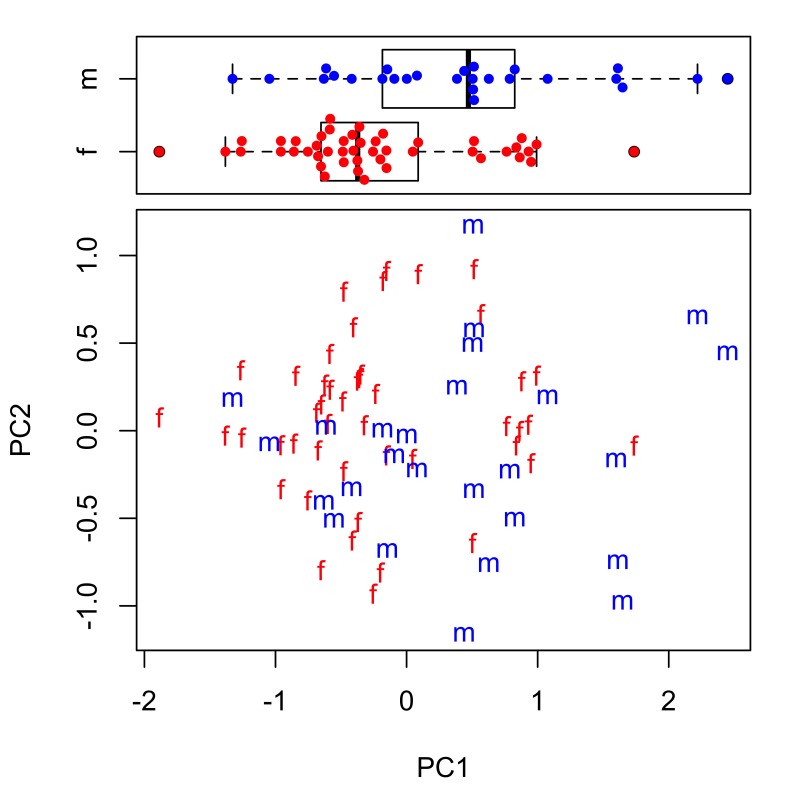
Scatterplot of the scores for the first two principal components (PC1 and PC2). Inset boxplot shows distribution of scores for PC1. Key: f, female; m, male.

**Figure 3b.**
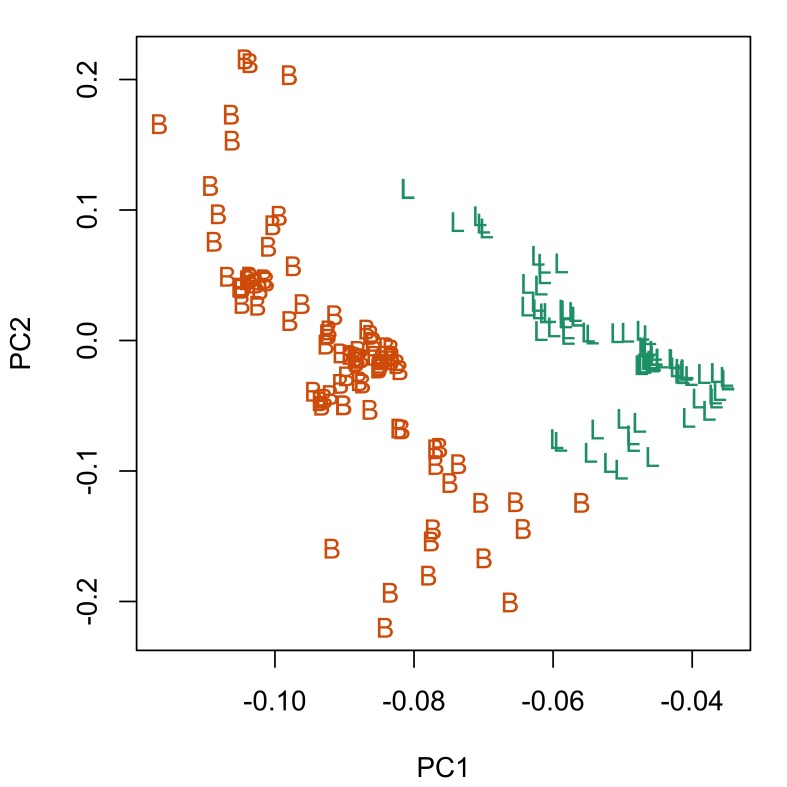
Scatterplot of the loadings for PC1 and PC2. Key: L, length variables; B, base variables.

**Figure 4a.**
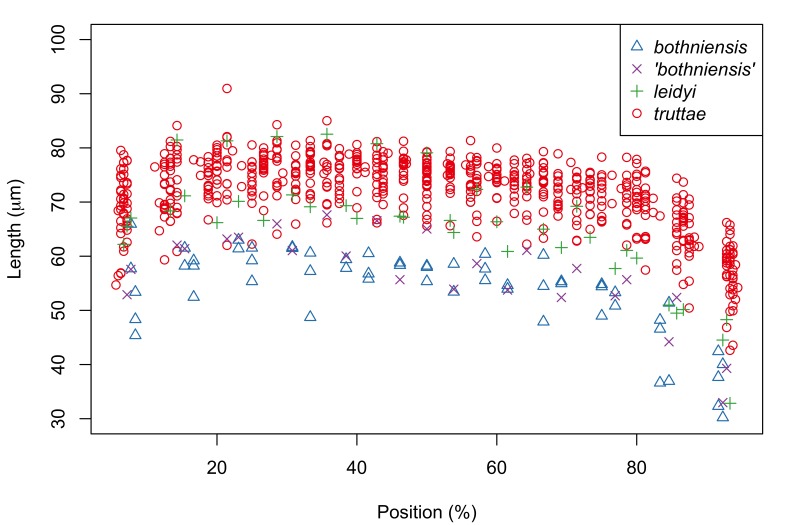
Hook blade length plotted against standardized position.

**Figure 4b.**
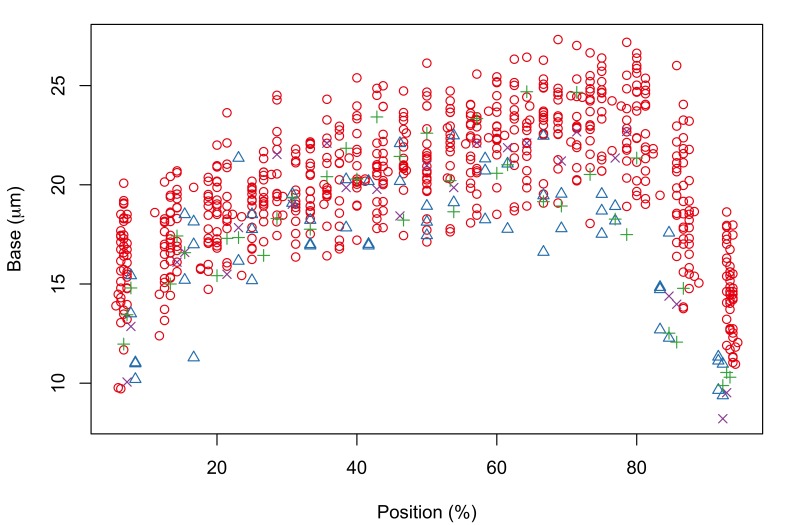
Hook base width plotted against standardized position.

**Figure 5a.**
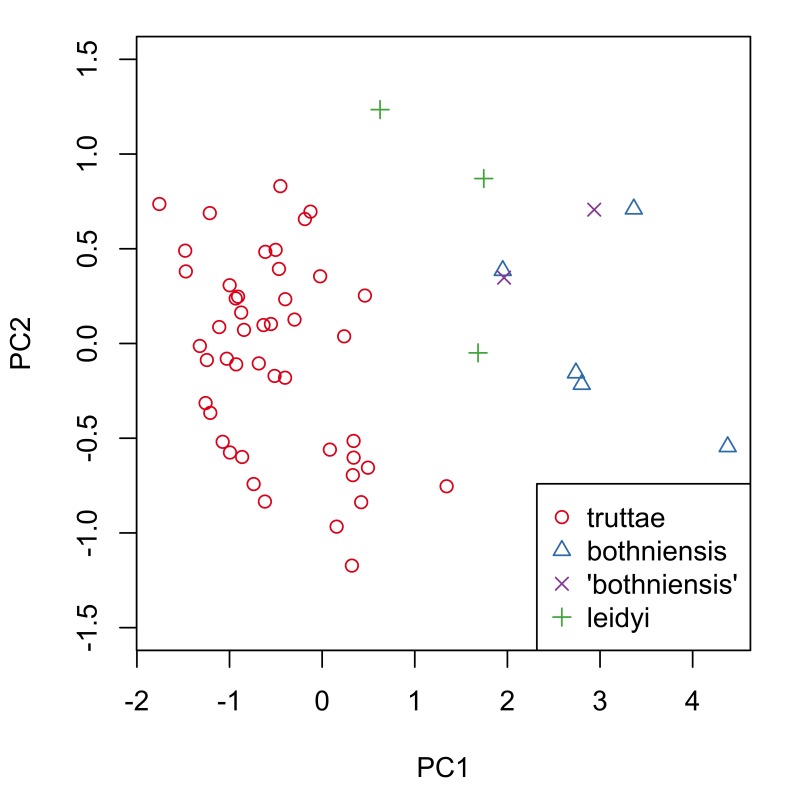
Scatterplot of the scores for the first two principal components (PC1 and PC2).

**Figure 5b.**
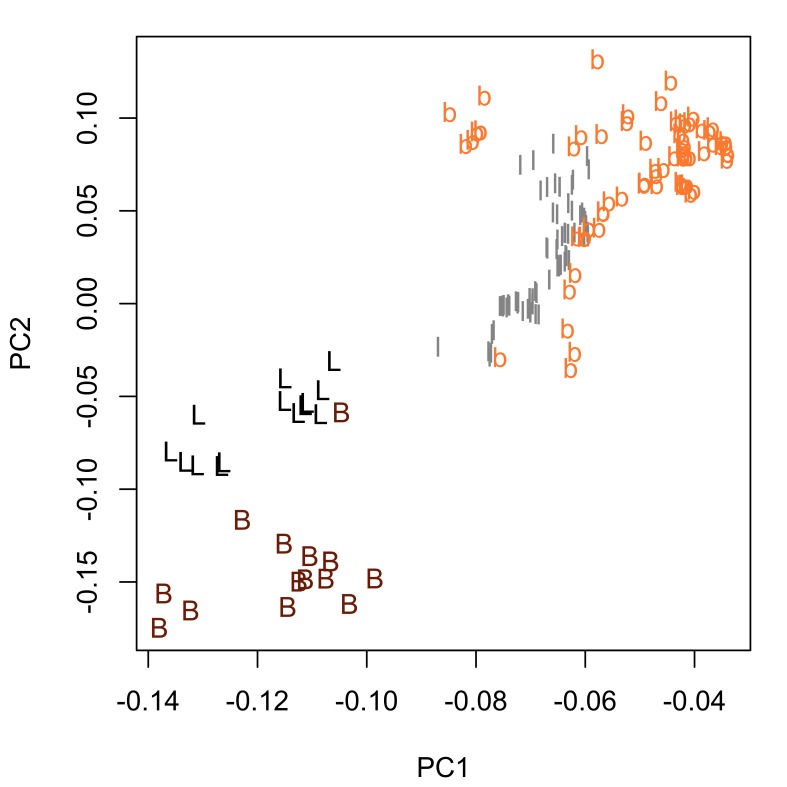
Scatterplot of the loadings for the first two principal components. Key: l and b, length and base measurements respectively, from hooks in the 4.5-79.5% region of the proboscis; L and B, length and base measurements respectively, from hooks in the 80.5-95.5% region of the proboscis.

**Figure 7a.**
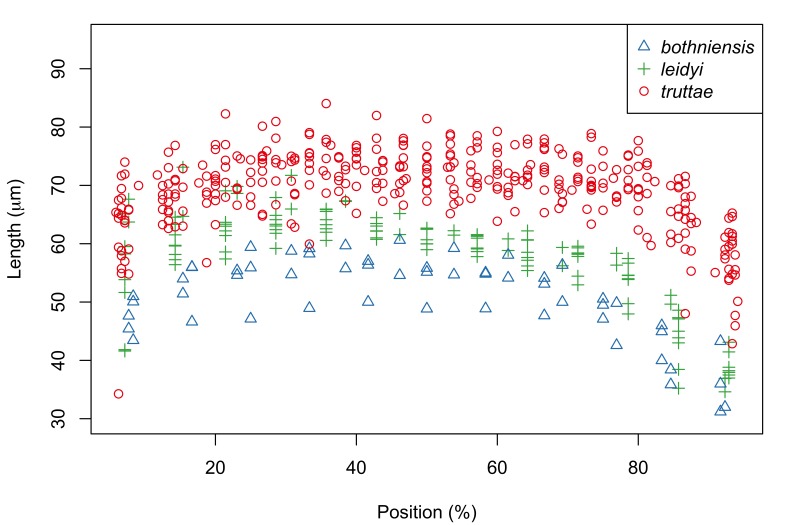
Hook blade length plotted against standardized position.

**Figure 7b.**
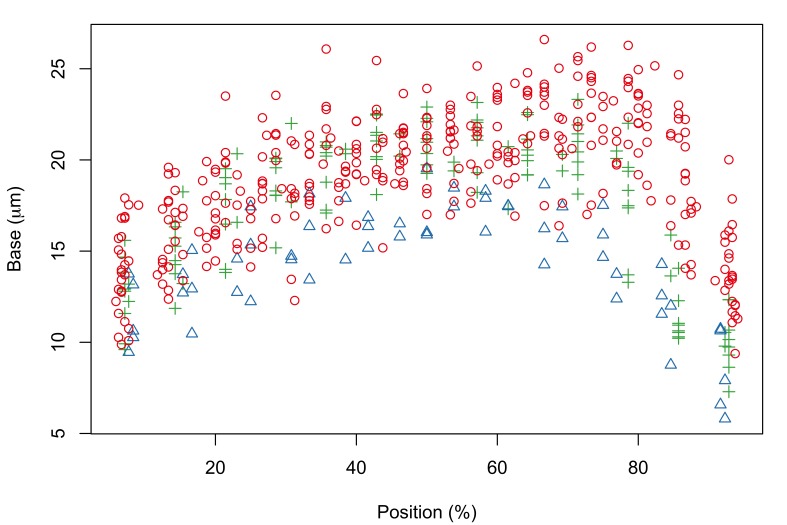
Hook base width plotted against standardized position.

**Figure 8a.**
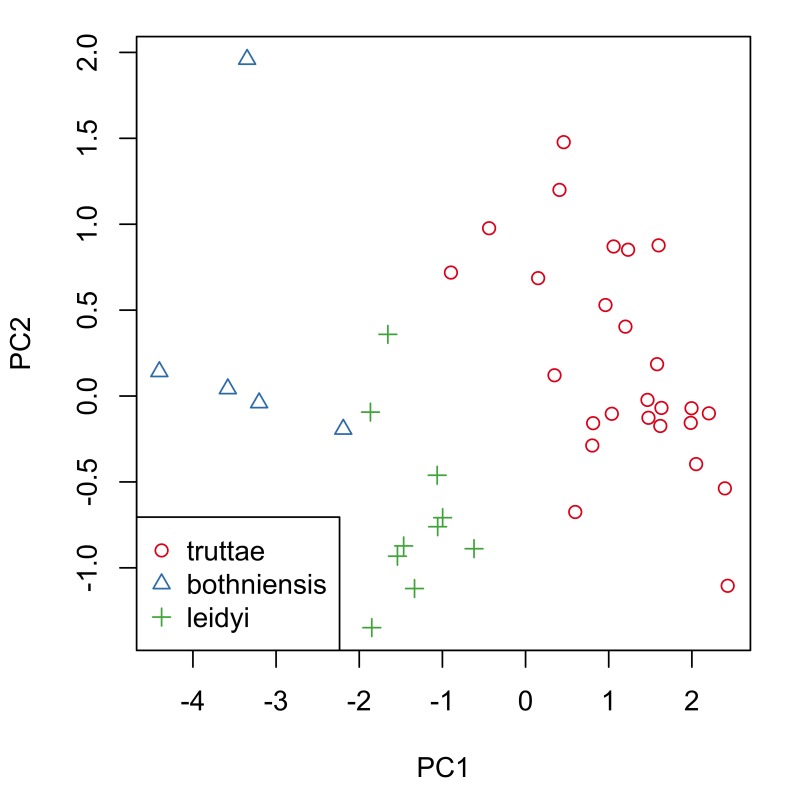
Scatterplot of the scores for the first two principal components (PC1 and PC2).

**Figure 8b.**
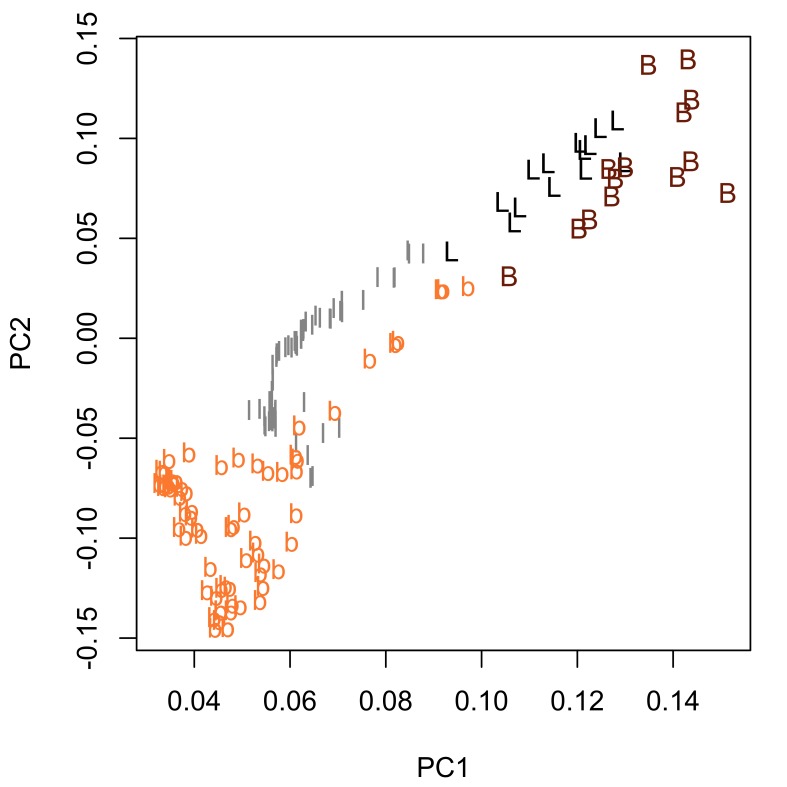
Scatterplot of the loadings for the first two principal components. Key: l and b, length and base measurements respectively, from hooks in the 5-79% region of the proboscis; L and B, length and base measurements respectively, from hooks in the 80-95% region of the proboscis.

**Figure 10a.**
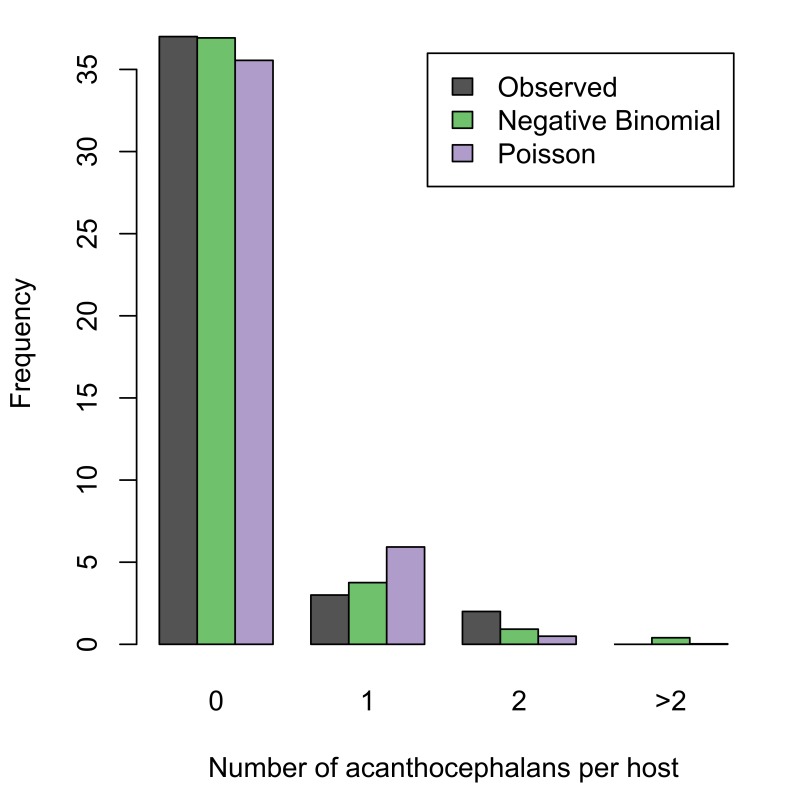
Loch Coulter Burn. Negative binomial distribution has parameters: mu=0.167 and k=0.261. Poisson distribution has parameter lambda=0.167. Akaike's information criterion (AIC) for fitted distributions: negative binomial, 43.3; Poisson, 43.9.

**Figure 10b.**
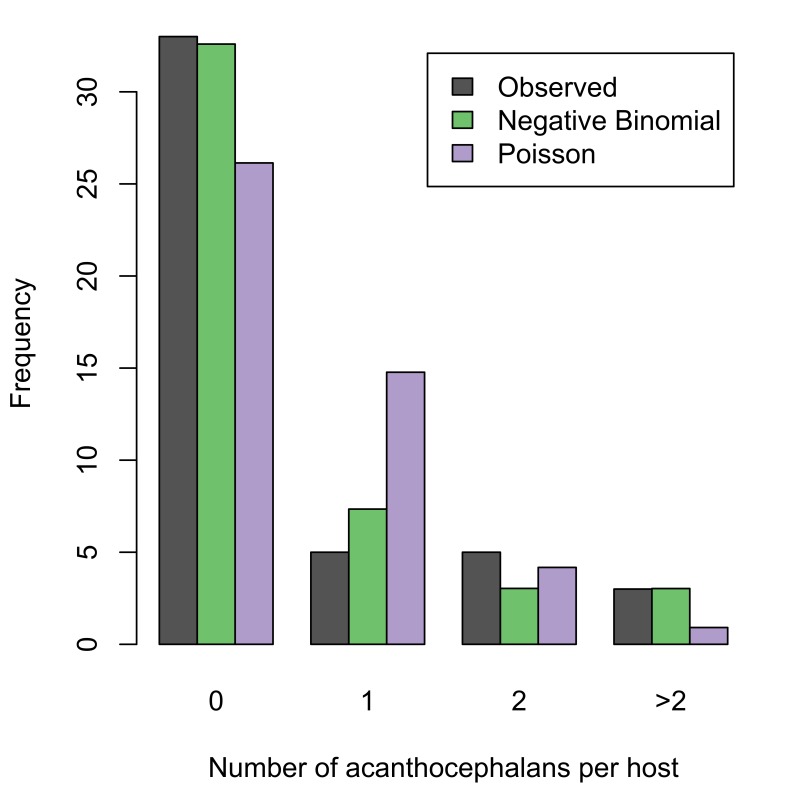
Loch Walton Burn. Negative binomial distribution has parameters: mu=0.565 and k=0.375. Poisson distribution has parameter lambda=0.565. AIC for fitted distributions: negative binomial, 97.5; Poission, 107.3.

**Table 1.** Material Studied.

Species	Host	Locality	Date Collected	Accession Numbers	ID Prefix in Supplementary Files	Number of Specimens
*Echinorhynchus truttae*	*Salmo trutta* L.	Drummore, southwest Scotland	NA	BM (NH) 1986.764–793	t1.	74 (45 f, 29 m)
*Echinorhynchus truttae*	*Salmo trutta*	Loch Walton Burn, River Carron catchment, central Scotland (National Grid Reference NS 668 865)	24th June 1996	BM (NH) 2002.2.4.264–275	t2.	11 (4 f, 7 m)
*Echinorhynchus truttae*	*Salmo trutta*	Loch Coulter Burn, River Carron catchment, central Scotland (National Grid Reference NS 761 865)	20th September 1996	BM (NH) 2002.2.4.276–283	t3.	8 (8 f , 0 m)
*Echinorhynchus bothniensis*	*Osmerus eperlanus* L.	Bothnian Bay, Baltic Sea	13th July 1985	BM (NH) 1987.1070–1074 (paratypes)	b1.	1 (1 f, 0 m)
*Echinorhynchus bothniensis*	*Osmerus eperlanus*	Lake Keitele, central Finland	10th October 1996	BM (NH) 2002.2.4.102–122	b2.	19 (8 f, 0 m)
*Echinorhynchus bothniensis*	*Osmerus eperlanus*	Lake Keitele, central Finland	26th October 1989	BM (NH) 1989.1474–1491	b4.	13 (6 f, 7 m)
*Echinorhynchus 'bothniensis'*	*Salvelinus alpinus* (L.)	Lake Pulmankijärvi, northern Finland	14th June 1989	BM (NH) 1989.1241–1248	b5.	7 (4 f, 3 m)
*Echinorhynchus 'bothniensis'*	*Salvelinus alpinus*	Lake Pulmankijärvi, northern Finland	NA	BM (NH) 1989.1439–1468	b6.	2 (2 f, 0 m)
*Echinorhynchus 'bothniensis'*	*Coregonus lavaretus* (L.)	Lake Pulmankijärvi, northern Finland	NA	BM (NH) 1989.1259–1270	b7.	16 (8 f, 8 m)
*Echinorhynchus 'bothniensis'*	*Coregonus lavaretus*	Lake Pulmankijärvi, northern Finland	14th–16th June 1989	BM (NH) 1989.1406–1420	b8.	5 (3 f, 2 m)
*Echinorhynchus 'bothniensis'*	*Platichthys flesus* (L.)	Lake Pulmankijärvi, northern Finland	11th June 1990	NA	b9.	4 (3 f, 1 m)
*Echinorhynchus leidyi*	*Salvelinus alpinus*	Kinguk Lake, Northwest Territories, Canada 64°40´N 75°30´W	27th August 1984	CMNPA 1985–0146	l1.	3 (3 f, 0 m)
*Echinorhynchus leidyi*	*Coregonus lavaretus*	Southern Indian Lake, Manitoba, Canada 58°45´N 98°55´W	8th June 1982	CMNPA 1985–0138	l2.	5 (0 f, 5 m)
*Echinorhynchus leidyi*	*Salvelinus alpinus*	Unnamed lake, Northwest Territories, Canada 64°26´N 77°45´W	29th August 1984	CMNPA 1985–0149	l3.	5 (0 f, 5 m)

**Table 2.** Morphometrics of female *Echinorhynchus bothniensis*, *Echinorhynchus 'bothniensis'*, *Echinorhynchus leidyi* and *Echinorhynchus truttae* (range; mean + standard deviation and sample size in parentheses). Data available in Suppl. materials 1, 3.

	*Echinorhynchus bothniensis* Bothnian Bay (Zdzitowiecki and Valtonen, 1987)	*Echinorhynchus bothniensis* Lake Keitele (this study)	*Echinorhynchus 'bothniensis'* Lake Pulmankijärvi (this study)	*Echinorhynchus leidyi* Northern Canada (Shostak et al., 1986)	*Echinorhynchus truttae* Scotland (this study)
Body length (mm)	10.5 – 27.1 (—; 38)	10.1 - 25.1 (16.0 ± 4.44; 14)	8.2 – 15.8 (10.9 ± 2.28; 18)	3.9 – 31.6 (16.4 ± 4.36; 476)	9.0 – 18.9 (14.0 ± 2.00; 56)
Body width (mm)	1.12 – 3.13 (—; 38)	1.14 – 2.76 (1.89 ± 0.50; 14)	0.71 – 2.72 (1.32 ± 0.50; 20)	0.60 – 3.0 (1.2 ± 0.26; 478)	0.85 – 2.02 (1.19± 0.25; 56)
Body length/width	—	5.6 – 11.8 (8.6 ± 1.52; 14)	3.8 – 13.8 (9.2 ± 2.34; 18)	4.3 – 27.4 (13.7 ± 3.40; 466)	7.4 – 16.5 (12.1 ± 2.02; 56)
Proboscis length	660 – 940 (846 ± 60; 38)	611 – 787 (717 ± 56.6; 7)	711 – 904 (823 ± 77.3; 5)	733 – 1335 (1037 ± 116.6; 508)	869 – 1188 (1009 ± 59.7; 56)
Proboscis width	230 – 290 (264 ± 15; 38)	248 – 344 (308 ± 33.2; 11)	213 – 334 (285 ± 34.3; 19)	187 – 355 (274 ± 31.0; 508)	249 – 359 (309 ± 22.2; 56)
Proboscis length/width	2.82 – 3.67 (3.21 ± 0.21; 38)	2.03 ± 2.95 (2.47 ± 0.370; 7)	2.61 – 3.77 (3.04 ± 0.500; 5)	2.64 – 5.98 (3.81 ± 0.414; 508)	2.73 – 3.93 (3.28 ± 0.289; 56)
Number of rows of hooks	18 – 22	18 – 21 (19.2 ± 0.98; 14)	18 – 22 (19.5 ± 1.07; 19)	14 – 23 (18.1 ± 1.66; 508)	16 – 22 (19.6 ± 1.44; 57)
Number of hooks per row	11 – 15	11 – 12 (11.9 ± 0.35; 8)	12 – 15 (13.2 ± 1.10; 5)	10 – 17 (14.1 ± 1.11; 508)	12 – 17 (14.6 ± 0.98; 57)
Maximum length of hook blade	57 – 72 (64 ± 3.0; 38)	57 – 66 (61 ± 3.6; 4)	64 – 68 (65 ± 2.1; 3)	52 – 84 (70 ± 4.8; 508)	68 – 91 (78 ± 3.8; 46)
Proboscis receptacle length	1080 – 1850 (1497 ± 176; 38)	1237 – 2195 (1615 ± 249; 14)	668 – 1922 (1284 ± 323; 20)	—	1486 – 2855 (1901 ± 287; 56)
Proboscis receptacle width	300 – 430 (366 ± 33; 38)	336 – 618 (436 ± 77; 14)	167 – 431 (296 ± 63; 20)	—	318 *±* 616 (407 ± 77; 56)
Lemniscus length	870 – 1890 (—; 38)	958 – 1963 (1462 ± 323; 14)	510 – 1543 (901 ± 290; 19)	—	935 – 2434 (1670 ± 293; 56)
Lemniscus width	220 – 540 (—; 38)	212 – 616 (361 ± 111; 14)	99 – 441 (266 ± 90; 19)	—	201 – 693 (350 ± 93; 56)
Genital complex length	1480 – 2270 (1846 ± 201; 38)	1575 – 2104 (1912 ± 186; 6)	991 – 1669 (1356 ± 193; 12)	—	1357 – 2761 (1792 ± 289; 25)
Uterine bell length	—	375 – 734 (551 ± 147; 6)	265 – 555 (368 ± 93; 12)	—	429 – 878 (568 ± 93; 25)
Uterus length	—	1060 – 1749 (1314 ± 212; 8)	646 – 1203 (902 ± 158; 13)	—	614 – 1592 (1003 ± 191; 42)
Uterus width	—	110 – 237 (161 ± 44.1; 11)	41 – 157 (71 ± 34.1; 16)	—	56 – 219 (110 ± 30.1; 55)
Vagina length	—	218 – 344 (273 ± 42.9; 14)	183 – 281 (221 ± 25.6; 14)	—	234 – 394 (294 ± 29.7; 56)
Vagina width	—	62 – 144 (103 ± 26.1; 14)	65 – 98 (80 ± 10.3; 14)	—	72 – 149 (109 ± 15.2; 56)
Vaginal sphincter width	—	97 – 208 (142 ± 33.9; 14)	61 – 125 (82 ± 19.3; 15)	—	91 – 182 (126 ± 19.4; 56)
Spincter width to vagina width ratio	—	1.04 – 1.97 (1.41 ± 0.271; 14)	0.73 – 1.28 (1.02 ± 0.184; 14)	—	0.88 – 2.01 (1.17 ± 0.161; 56)
Egg length	140 – 168 (156 ± 7; 38)	127 – 166 (148 ± 12.6; 15)	121 – 152 (137 ± 11.4; 9)	90 – 135 (115 ± 8.2; 134)	120 – 173 (140 ± 11.0; 117)
Egg width	22 – 29 (25 ± 1; 38)	19 – 31 (23 ± 3.1; 15)	19 – 23 (21 ± 1.2; 9)	—	22 – 34 (27 ± 2.2; 117)
Acanthor length	—	67 –80 (73 ± 3.5; 15)	67 – 78 (74 ± 3.9; 9)	—	70 – 90 (80 ± 4.4; 117)
Acanthor width	—	14 – 19 (17 ± 1.5; 15)	14 – 19 (17 ± 1.5; 9)	—	17 – 24 (20 ± 1.4; 117)

**Table 3.** Morphometrics of male *Echinorhynchus bothniensis*, *Echinorhynchus 'bothniensis'*, *Echinorhynchus leidyi* and *Echinorhynchus truttae* (range; mean + standard deviation and sample size in parentheses). Data available in Suppl. material 2.

	*Echinorhynchus bothniensis* Bothnian Bay (Zdzitowiecki and Valtonen, 1987)	*Echinorhynchus bothniensis* Lake Keitele (this study)	*Echinorhynchus 'bothniensis'* Lake Pulmankijärvi (this study)	*Echinorhynchus leidyi* Northern Canada (Shostak et al., 1986)	*Echinorhynchus truttae* Scotland (this study)
Body length (mm)	8.9 – 15.8	7.4 – 15.9 (10.9 ± 2.9; 16)	4.5 – 9.7 (7.3 ± 1.6; 14)	5.1 – 19.7 (10.3 ± 2.51; 360)	7.2 – 10.9 (8.9 ± 1.09; 32)
Body width (mm)	1.13 – 2.39	0.93 – 2.17 (1.47 ± 0.36; 14)	0.58 – 1.78 (1.04 ± 0.37; 14)	0.6 – 1.9 (1.0 ± 0.20; 353)	0.69 – 1.32 (0.90 ± 0.12; 32
Body length/width	—	5.5 – 10.3 (7.8 ± 1.42; 14)	4.9 – 10.2 (7.4 ± 1.40; 14)	5.6 – 21.0 (10.7 ± 3.03; 352)	6.7 – 12.2 (10.0 ± 1.29; 32)
Reproductive system length (mm)	—	5.1 – 11.0 (7.4 ± 2.17; 13)	3.0 – 6.3 (4.8 ± 1.08; 14)	—	4.0 – 6.6 (5.4 ± 0.69; 32)
Proboscis length	690 – 830 (756 ± 36; 50)	617 – 751 (683 ± 42.8;13)	—	658 – 1203 (930 ± 93.3; 381)	733 – 1019 (903 ± 59.6; 32)
Proboscis width	220 – 280 (240 ± 13; 50)	204 – 329 (265 ± 37.8; 16)	204 – 287 (256 ± 24.6; 8)	176 – 314 (245 ± 27.6; 381)	205 – 326 (264 ± 29.0; 32)
Proboscis length/width	2.69 – 3.51 (3.16 ± 0.22; 50)	2.00 – 3.16 (2.51 ± 0.327; 13)	—	2.57 – 5.24 (3.83 ± 0.424; 381)	2.67 – 4.07 (3.46 ± 0.381; 32)
Number of rows of hooks	17 – 20	17 – 21 (19.0 ± 1.50; 17)	18 – 22 (19.4 ± 1.26 10)	12 – 22 (17.5 ± 1.77; 381)	16 – 22 (18.7 ± 1.45; 35)
Number of hooks per row	11 – 14	11 – 13 (11.9 ± 0.59; 15)	—	10 – 16 (13.4 ± 0.98; 381)	11 – 15 (14.0 ± 0.95; 35)
Maximum length of hook blade	55 – 71 (62 ± 4; 50)	50 – 61 (57 ± 3.9; 6)	—	45 – 82 (64 ± 4.8; 381)	67 – 84 (75 ± 3.7; 26)
Proboscis receptacle length	1140 – 1800 (1452 ± 137; 50)	1042 – 1982 (1559 ± 231; 17)	913 – 1262 (1086 ± 125; 13)	—	1376 – 2384 (1779 ± 199; 32)
Proboscis receptacle width	240 – 350 (303 ± 27; 50)	141 – 402 (332 ± 67; 17)	154 – 345 (257 ± 62.6; 14)	—	278 – 499 (369 ± 41.9; 32)
Lemniscus length	720 – 1470	756 – 1678 (1219 ± 281; 15)	496 – 977 (717 ± 157; 11)	—	1172 – 1775 (1468 ± 164; 32)
Lemniscus width	150 – 480	173 – 553 (326 ± 106;15)	107 – 268 (207 ± 54.3; 12)	—	135 – 390 (288 ± 58.3; 32)
Anterior testes length	800 – 1680	761 – 1682 (1172 ± 332; 12)	403 – 934 (649 ± 165; 13)	—	707 – 1249 (1050 ± 126; 28)
Anterior testes width	370 – 670	289 – 831 (476 ± 145; 12)	136 – 447 (312 ± 88.0; 13)	—	394 – 637 (513 ± 70.0; 28)
Posterior testes length	810 – 1700	686 – 1602 (1069 ± 295; 12)	387 – 929 (640 ± 161; 13)	—	694 – 1198 (975 ± 136; 28)
Posterior testes width	300 – 680	306 – 837 (475 ± 158; 12)	197 – 471 (334 ± 84; 13)	—	394 – 591 (506 ± 55; 28)
Cement gland width	—	178 – 954 (356 ± 207; 17)	164 – 404 (282 ± 84; 14)	—	198 – 575 (365 ± 83; 32)
Saefftigen´s pouch length	750 – 1050	659 – 1413 (925 ± 227; 17)	500 – 871 (684 ± 117; 13)	—	538 – 854 (733 ± 77; 32)
Saefftigen´s pouch width	160 – 270	116 – 336 (227 ± 72; 17)	101 – 237 (165 ± 45; 13)	—	187 – 374 (288 ± 44; 32)
Penis width	85 – 113 (98 ± 7; 50)	50 – 105 (79 ± 16; 16)	45 – 89 (63 ± 12; 9)	—	66 – 110 (85 ± 11; 32)
Bursal sucker diameter	—	137 – 219 (182 ± 23; 11)	135 – 191 (164 ± 16; 10)	—	123 – 197 (152 ± 20; 15)

**Table 4.** **Correlation of morphometric variables with body length in female *Echinorhynchus truttae*.** Correlation measured by Pearson's product-moment correlation coefficient (r). The raw p value is the probability that the sample correlation coefficient could have come from a population with a correlation coefficient of zero. The Bonferroni correction was used to control the family wise error rate across multiple tests of significance. Data available in Suppl. material 1.

Variable	n	r	raw p	Bonferroni p
Body width	56	0.507	0.000066	**0.000997**
Proboscis length	56	0.563	0.000006	**0.000092**
Proboscis width	56	0.041	0.763773	1.000000
Proboscis receptacle length	56	0.533	0.000023	**0.000346**
Proboscis receptacle width	56	0.375	0.004442	0.066630
Lemniscus length	56	0.603	<0.000001	**0.000013**
Lemniscus width	56	0.487	0.000142	**0.002128**
Genital complex length	25	0.438	0.028697	0.430462
Uterine bell length	25	0.266	0.198106	1.000000
Uterus length	42	0.376	0.014200	0.212997
Uterus width	55	0.123	0.369147	1.000000
Vagina length	56	0.273	0.041850	0.627757
Vagina width	56	0.496	0.000100	**0.001500**
Vaginal sphincter width	56	0.501	0.000085	**0.001281**
Maximum length of hook blade	46	0.267	0.072923	1.000000

**Table 5.** **Correlation of morphometric variables with body length in male *Echinorhynchus truttae*.** Correlation measured by Pearson's product-moment correlation coefficient (r). The raw p value is the probability that the sample correlation coefficient could have come from a population with a correlation coefficient of zero. The Bonferroni correction was used to control the family wise error rate across multiple tests of significance. Data available in Suppl. material 2.

Variable	n	r	raw p	Bonferroni p
Reproductive system length	32	0.936	<0.000001	**<0.000001**
Body width	32	0.417	0.017468	0.314424
Proboscis length	32	0.298	0.097440	1.000000
Proboscis width	32	-0.054	0.769724	1.000000
Proboscis receptacle length	32	0.131	0.474205	1.000000
Proboscis receptacle width	32	0.236	0.193402	1.000000
Lemniscus length	32	0.698	0.000009	**0.000159**
Lemniscus width	32	0.330	0.064692	1.000000
Anterior testis length	28	0.588	0.001008	**0.018152**
Anterior testis width	28	0.446	0.017358	0.312447
Posterior testis length	28	0.685	0.000059	**0.001058**
Posterior testis width	28	0.352	0.065541	1.000000
Cement gland width	32	0.296	0.099633	1.000000
Saefftigen´s pouch length	32	0.360	0.043181	0.777265
Saefftigen´s pouch width	32	0.174	0.339571	1.000000
Penis width	32	0.217	0.232671	1.000000
Bursal sucker diameter	15	0.259	0.350967	1.000000
Maximum length of hook blade	23	0.428	0.041548	0.747868

**Table 6.** Cement gland arrangement in males of the *Echinorhynchus bothniensis* group and *Echinorhynchus truttae*. Notation for cement gland pattern from Shostak et al. (1986): B, clumped, three staggered pairs; C, chainlike, two pairs and two singles; D, chainlike, one pair and four singles; E, chainlike, six singles. Only specimens with six cement glands included. Data available in Suppl. material 2.

	B	C	D	E
*Echinorhynchus bothniensis* (Lake Keitele)	1	4	10	4
(5.30%)	(21.10%)	(52.60%)	(21.10%)
*Echinorhynchus 'bothniensis'* (Lake Pulmankijärvi)	0	0	4	5
(44.40%)	(55.60%)
*Echinorhynchus leidyi* (Northern Canada, Shostak et al., 1986)	1	36	181	118
(0.30%)	(10.70%)	(53.90%)	(35.10%)
*Echinorhynchus truttae* (Scotland)	1	16	13	0
(3.30%)	(53.30%)	(43.30%)

**Table 7.** **Frequency distribution of *Echinorhynchus truttae* in definitive host populations.** 95% confidence limits (where applicable) in parentheses. Data available in Suppl. materials 6, 7.

	Loch Coulter Burn	Loch Walton Burn
Number of fish examined	42	46
Prevalence (%)	0.119 (0.048 – 0.259)	0.283 (0.171 – 0.434)
Mean intensity of infection	1.4 (1.0 – 1.6)	2 (1.46 – 2.69)
Maximum intensity of infection	2	5
Mean abundance	0.167 (0.0476 – 0.333)	0.565 (0304 – 0.935)
Overdispersion index (variance/mean)	1.44	2.1

## Supplementary files

### Standard morphometric and meristic data from females.

**Authors:** Matthew T Wayland **Data type:** morphological and meristic Comma separated value (csv) file of morphometric data from females. Rows are specimens and columns (column three onwards) are morphometric variables (*e.g.* proboscis length) or meristic variables (*e.g.* number of longitudinal rows of hooks). All morphometric measurements are in micrometres. The first column is species and the second column is a unique identifier for the specimen. The unique identifier is composed of two parts: the part before the full stop indicates the sample (please see table 1); the number after the full stop indicates the specimen number. In the species column, *Echinorhynchus bothniensis* and *Echinorhynchus 'bothniensis'* are listed as bothniensis1 and bothniensis2, respectively. **File name:** female_morphometrics.csv

### Standard morphometric and meristic data from males.

**Authors:** Matthew T Wayland **Data type:** morphological and meristic Comma separated value (csv) file of morphometric data from males. Rows are specimens and columns (column three onwards) are morphometric variables (*e.g.* proboscis length) or meristic variables (*e.g.* number of longitudinal rows of hooks). All morphometric measurements are in micrometres. The first column is species and the second column is a unique identifier for the specimen. The unique identifier is composed of two parts: the part before the full stop indicates the sample (please see table 1); the number after the full stop indicates the specimen number. In the species column *Echinorhynchus bothniensis* and *Echinorhynchus 'bothniensis'* are listed as bothniensis1 and bothniensis2, respectively. Notation for cement gland pattern from Shostak *et al.* (1986): B, clumped, three staggered pairs; C, chainlike, two pairs and two singles; D, chainlike, one pair and four singles; E, chainlike, six singles. **File name:** 2983.csv

### Egg and acanthor dimensions

**Authors:** Matthew T Wayland **Data type:** morphological Comma separated value file with 6 columns: species, specimen, egg length, acanthor length, egg width, acanthor width. All measurements in micrometres. The unique identifier for specimen is composed of two parts: the part before the full stop indicates the sample (please see table 1); the number after the full stop indicates the specimen number. Three eggs were measured from each gravid female. In the species column *Echinorhynchus bothniensis* and *Echinorhynchus 'bothniensis'* are listed as bothniensis1 and bothniensis2, respectively. **File name:** 3365.csv

### Hook measurement data from females

**Authors:** Matthew T Wayland **Data type:** morphological The file is a comma separated value (CSV) format suitable for input to the Acanthocephalan Proboscis Profiler software (http://acanthocephala.sourceforge.net). It includes data from one of the paratypes of *Echinorhynchus bothniensis* from the Bothnian Bay, Baltic Sea (specimen: b1.01). The file has 5 columns: specimen, group, hook, length and base. specimen - unique identifier for the specimen group - name of group (*Echinorhynchus bothniensis* and *Echinorhynchus 'bothniensis'* are listed as bothniensis1 and bothniensis2, respectively) hook - numerical position of hook in longitudinal row as counted from the distal end of the probocis length - length of hook blade (micrometres) base - width of hook base (micrometres) **File name:** 2743.csv

### Hook measurement data from males

**Authors:** Matthew T Wayland **Data type:** morphological The file is a comma separated value (CSV) format suitable for input to the Acanthocephalan Proboscis Profiler software (http://acanthocephala.sourceforge.net). The file has 5 columns: specimen, group, hook, length and base. specimen - unique identifier for the specimen group - name of group (following convention used in other data files, *Echinorhynchus bothniensis* is listed as bothniensis1) hook - numerical position of hook in longitudinal row as counted from the distal end of the probocis length - length of hook blade (micrometres) base - width of hook base (micrometres) **File name:** 2696.csv

### Frequency distribution of Echinorhynchus truttae in Salmo trutta from Loch Coulter Burn

**Authors:** Matthew T Wayland **Data type:** ecological Comma-separated value (CSV) file with two columns: host fork length (mm) and number of worms. Host fish were sampled from Loch Coulter Burn (National Grid Reference NS 761 865) on 20/9/1996. Acanthocephalan voucher specimens: BM(NH) 2002.2.4.276-283. **File name:** 3001.csv

### Frequency distribution of Echinorhynchus truttae in Salmo trutta from Loch Walton Burn

**Authors:** Matthew T Wayland **Data type:** ecological Comma-separated value (CSV) file with two columns: host fork length (mm) and number of worms. Host fish were sampled from Loch Walton Burn (National Grid Reference NS 668 865) on 24/6/1996. Acanthocephalan voucher specimens: BM(NH) 2002.2.4.264-275. **File name:** 3000.csv

### Boxplots showing sexual dimorphism in morphometric and meristic characters in Echinorhyhus truttae

**Authors:** Matthew T Wayland **Data type:** morphological Boxplots showing sexual dimorphism in morphometric and meristic data for Echinorhynchus truttae. For numbers specimens in each plot please see tables 2 and 3. **File name:** 3026.pdf

### Boxplots of morphometric and meristic data from female acanthocephalans.

**Authors:** Matthew T Wayland **Data type:** morphological Boxplots of morphometric and meristic data from female *Echinorhynchus bothniensis* (Lake Keitele), *Echinorhynchus 'bothniensis'* and *Echinorhynchus truttae*. **File name:** 3016.pdf

### Boxplots of morphometric and meristic data from male acanthocephalans.

**Authors:** Matthew T Wayland **Data type:** morphological Boxplots of morphometric and meristic data from male *Echinorhynchus bothniensis* (Lake Keitele), *Echinorhynchus 'bothniensis'* and *Echinorhynchus truttae*. **File name:** 3017.pdf
