# Supplementary figures and images for: Morphological variation in Echinorhynchus truttae Schrank, 1788 and the Echinorhynchus bothniensis Zdzitowiecki & Valtonen, 1987 species complex from freshwater fishes of northern Europe
Source: Biodivers Data J. 2013 Sep 16;(1):e975. doi: 10.3897/BDJ.1.e975 (PMC3964692; doi:10.3897/BDJ.1.e975)

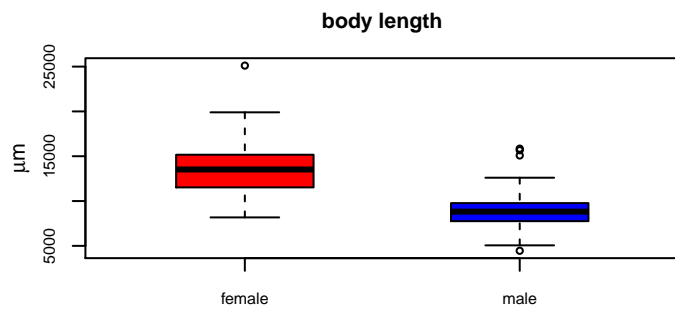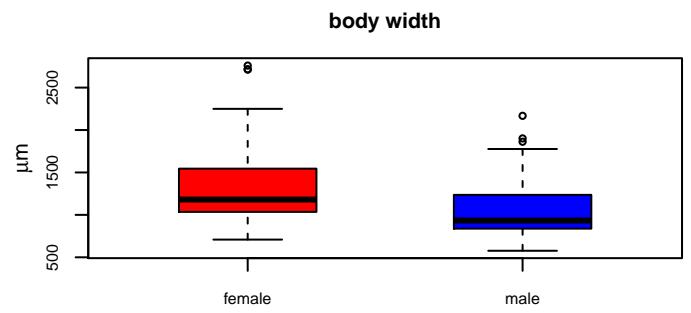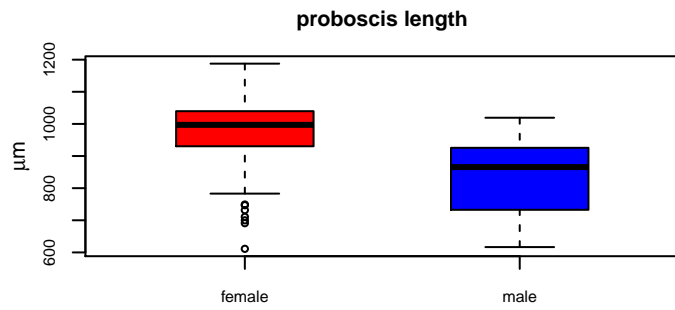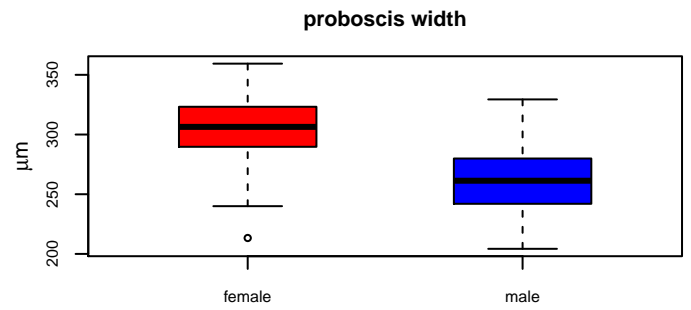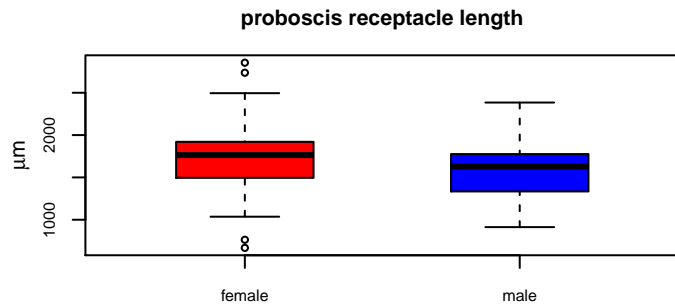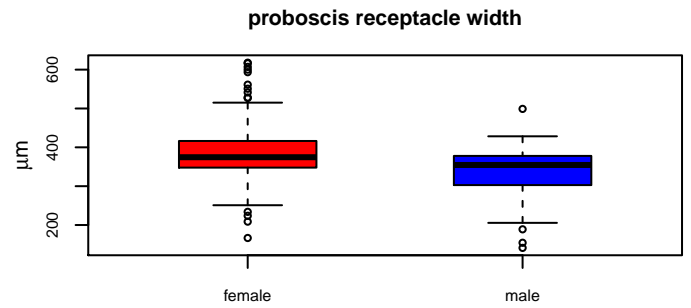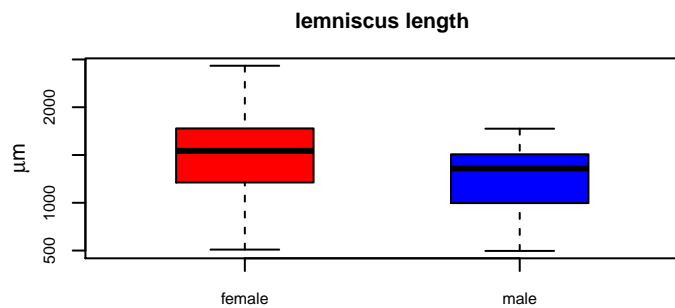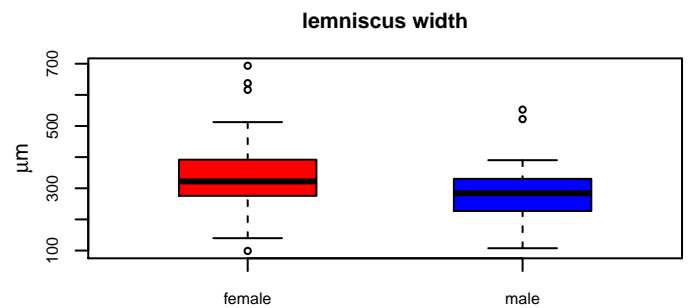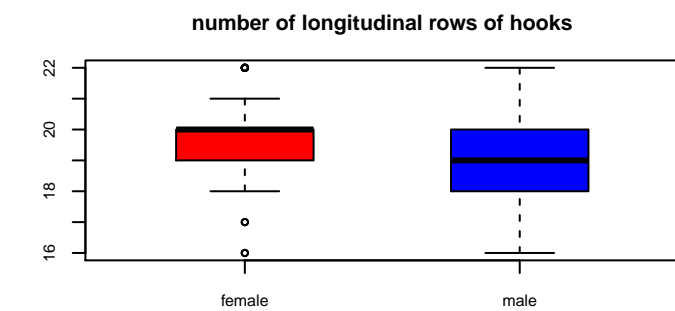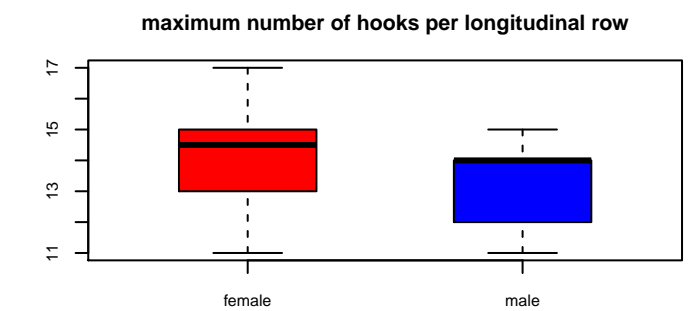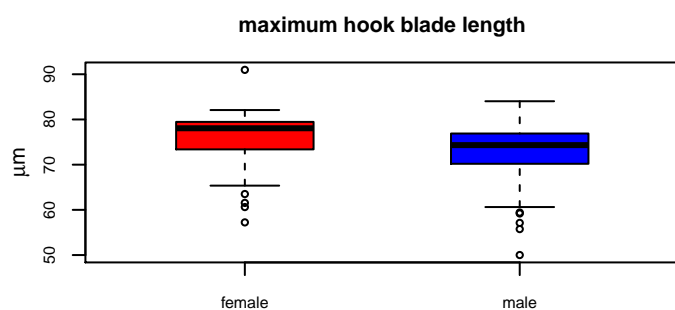

Supplement: Supplementary file 8 — Authors: Matthew T Wayland Data type: morphological Boxplots showing sexual dimorphism in morphometric and meristic data for Echinorhynchus truttae. For numbers specimens in each plot please see tables 2 and 3. File name: 3026.pdf [file biodiversity_data_journal-1-e975-s008.pdf]

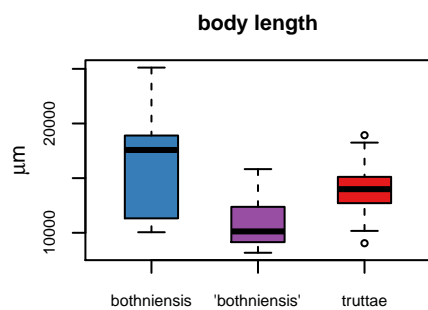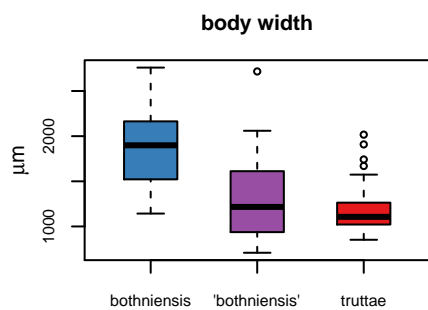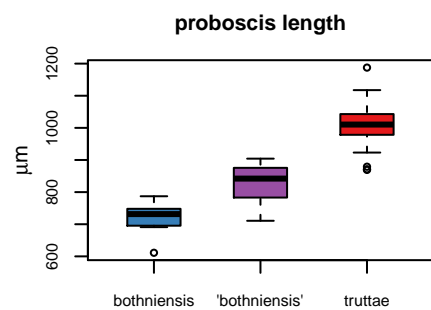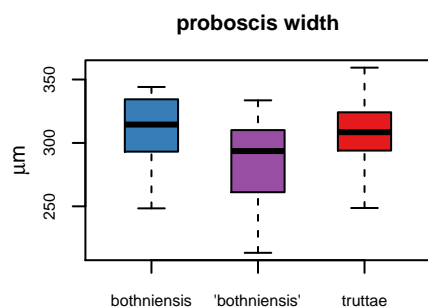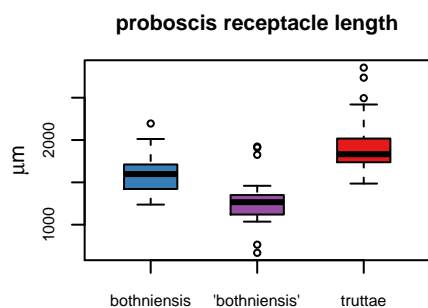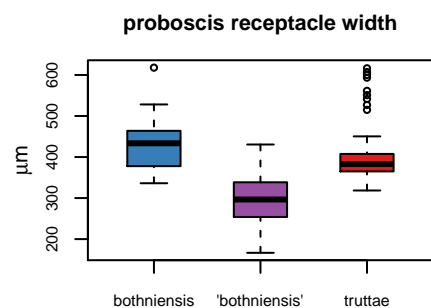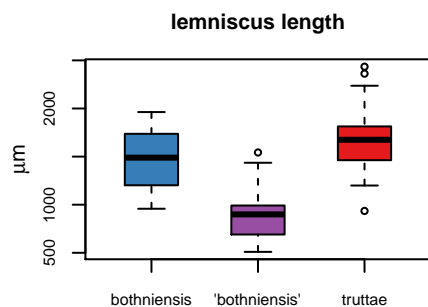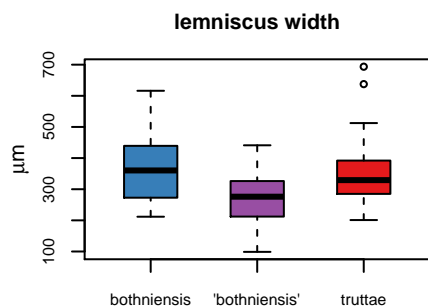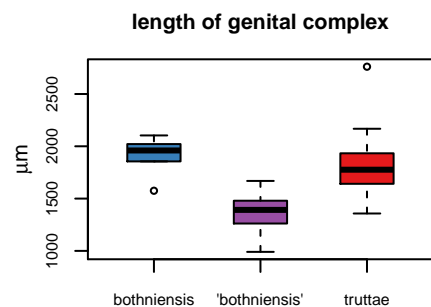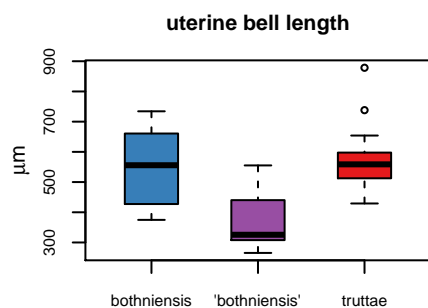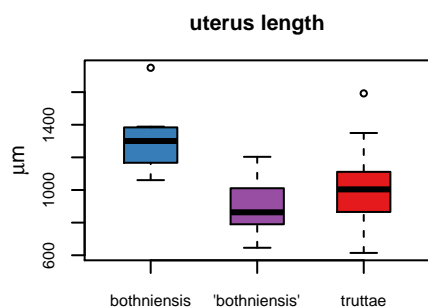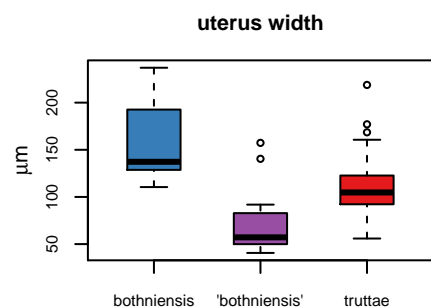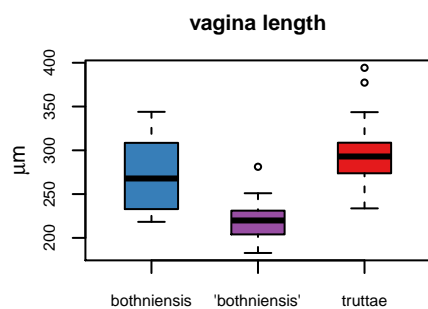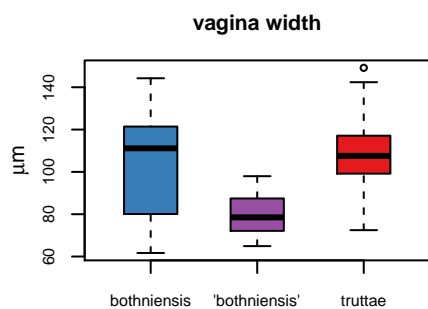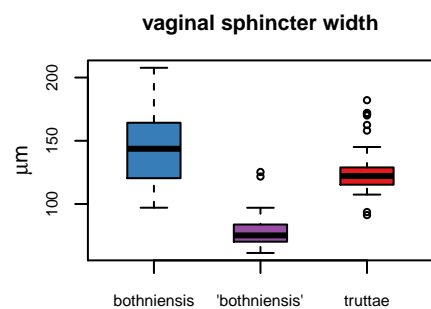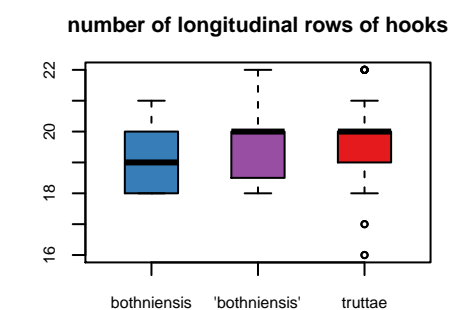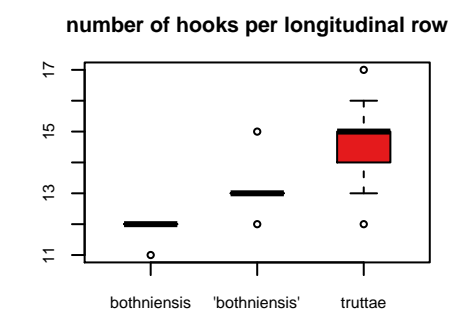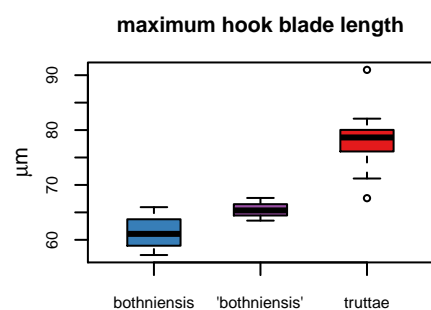

Supplement: Supplementary file 9 — Authors: Matthew T Wayland Data type: morphological Boxplots of morphometric and meristic data from female Echinorhynchus bothniensis (Lake Keitele), Echinorhynchus 'bothniensis' and Echinorhynchus truttae. File name: 3016.pdf [file biodiversity_data_journal-1-e975-s009.pdf]

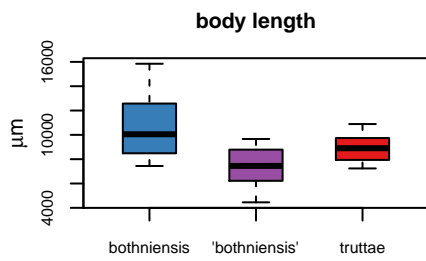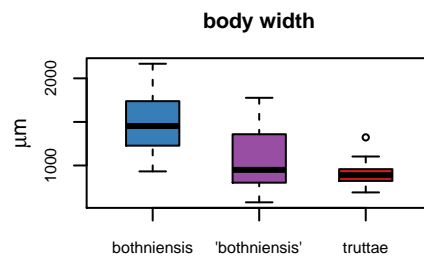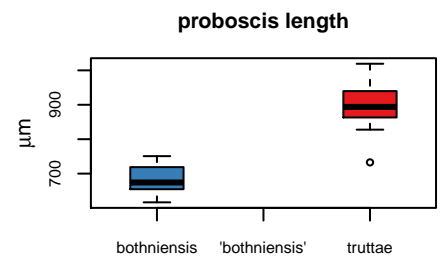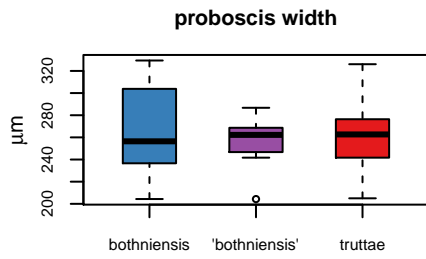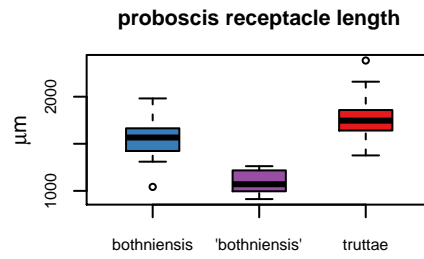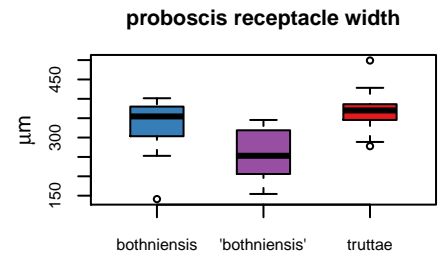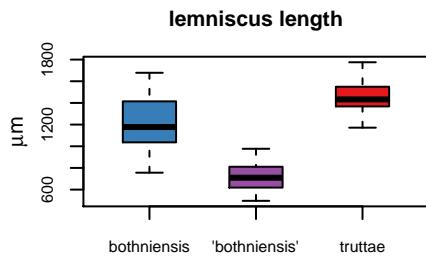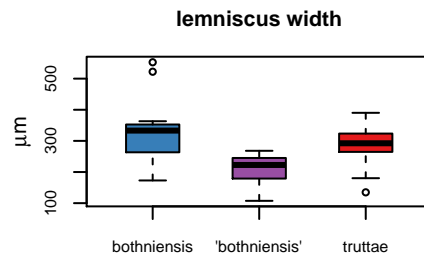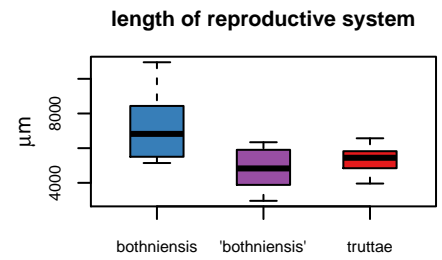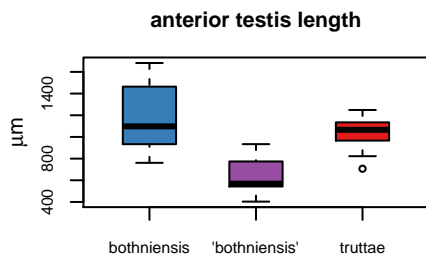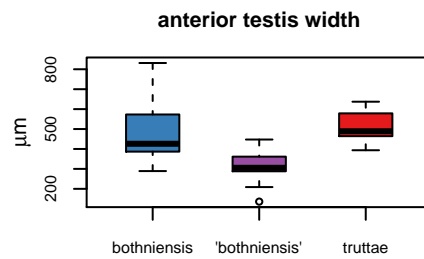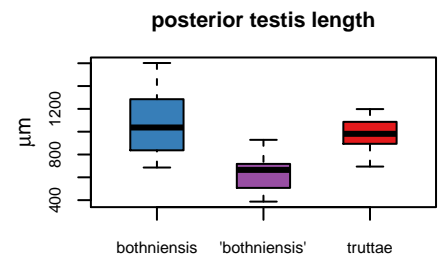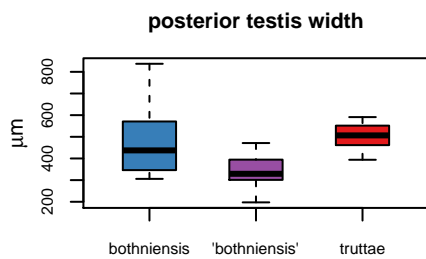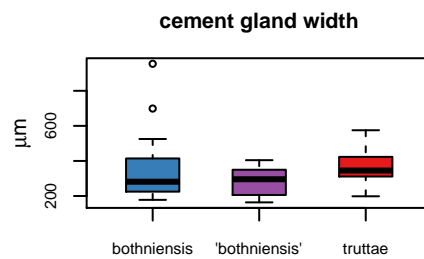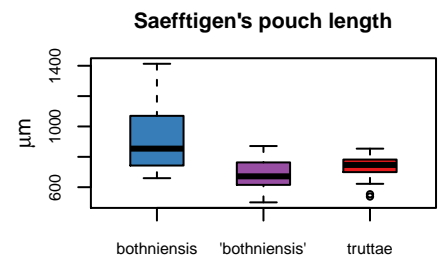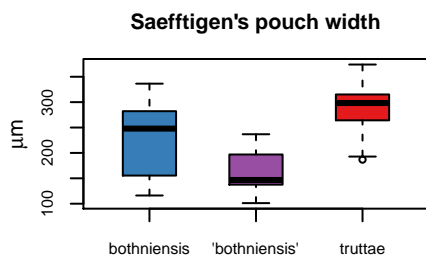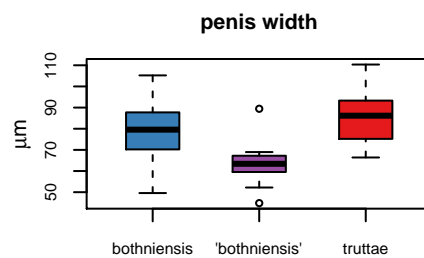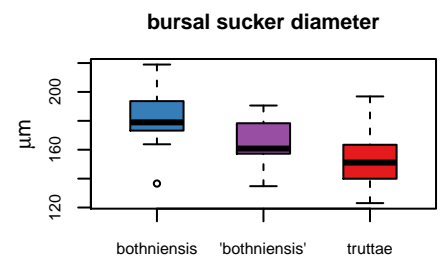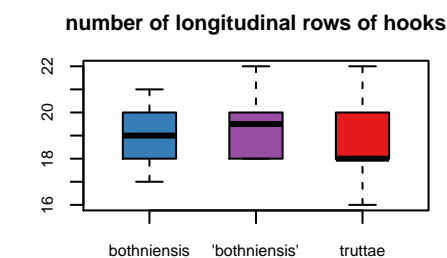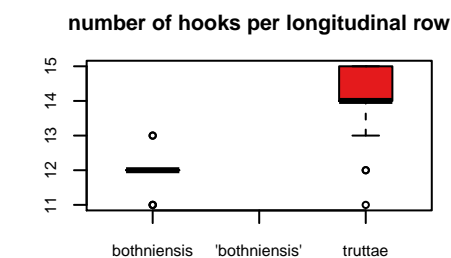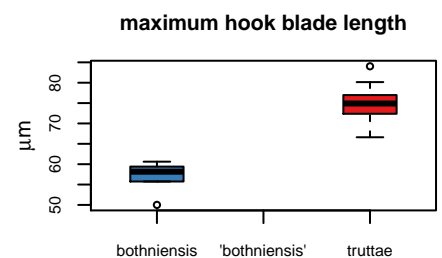

Supplement: Supplementary file 10 — Authors: Matthew T Wayland Data type: morphological Boxplots of morphometric and meristic data from male Echinorhynchus bothniensis (Lake Keitele), Echinorhynchus 'bothniensis' and Echinorhynchus truttae. File name: 3017.pdf [file biodiversity_data_journal-1-e975-s010.pdf]
